# Development of a Mechanistic Hypothesis Linking Compensatory Biomechanics and Stepping Asymmetry during Gait of Transfemoral Amputees

**DOI:** 10.1155/2019/4769242

**Published:** 2019-02-03

**Authors:** Abeer Mohamed, Andrew Sexton, Kirsten Simonsen, Chris A. McGibbon

**Affiliations:** ^1^Institute of Biomedical Engineering, University of New Brunswick, Fredericton, New Brunswick, Canada; ^2^Department of Mechanical Engineering, University of New Brunswick, Fredericton, New Brunswick, Canada; ^3^Eastern Prosthetic Clinic, Moncton, New Brunswick, Canada; ^4^Faculty of Kinesiology, University of New Brunswick, Fredericton, New Brunswick, Canada

## Abstract

**Objective:**

Gait asymmetry is a common adaptation observed in lower-extremity amputees, but the underlying mechanisms that explain this gait behavior remain unclear for amputees that use above-knee prostheses. Our objective was to develop a working hypothesis to explain chronic stepping asymmetry in otherwise healthy amputees that use above-knee prostheses.

**Methods:**

Two amputees (both through-knee; one with microprocessor knee, one with hydraulic knee) and fourteen control subjects participated. 3D kinematics and kinetics were acquired at normal, fast, and slow walking speeds. Data were analyzed for the push-off and collision limbs during a double support phase. We examined gait parameters to identify the stepping asymmetry then examined the external work rate (centre of mass) and internal (joint) power profiles to formulate a working hypothesis to mechanistically explain the observed stepping asymmetry.

**Results:**

Stepping asymmetry at all three gait speeds in amputees was characterized by increased stance phase duration of the intact limb versus relatively normal stance phase duration for the prosthesis limb. The prosthesis limb contributed very little to positive and negative work during the double support phase of gait. To compensate, the intact leg at heel strike first provided aid to the deficient prosthetic ankle/foot during its push-off by doing positive work with the intact knee, which caused a delayed stance phase pattern. The resulting delay in toe-off of the intact limb then facilitated the energy transfer from the more robust intact push-off limb to the weaker colliding prosthesis side. This strategy was observed for both amputees.

**Conclusions:**

There is a sound scientific rationale for a mechanistic hypothesis that stepping asymmetry in amputee participants is a result of a motor adaptation that is both facilitating the lower-leg trajectory enforced by the prosthesis while compensating for the lack of work done by the prosthesis, the cost of which is increased energy expenditure of the intact knee and both hips.

## 1. Introduction

It is well documented that users of above-knee prostheses have persistent gait abnormalities [1–3], with increased gait asymmetry [4–6] and increased energy expenditure [7–9] being two of the hallmark features of amputee gait. Transfemoral amputees have more falls than their age-matched peers [10], have a significantly higher risk of developing osteoarthritis of the intact knee and/or hips [11], and are more likely to become sedentary which contributes to declining health and quality of life [12].

How unilateral amputees biomechanically compensate for their prosthesis has been studied for decades [2, 3]. Whether below- or above-knee, one of the most common characteristics of amputee gait is the reduction of push-off power of the artificial foot in terminal stance, requiring the hip of the amputee's residual limb to compensate for this deficiency [7, 13–15]. Another common finding among studies is the asymmetric stepping pattern, typically characterized as a longer stance phase duration of the intact limb, compared to the prosthetic limb [1, 16–18].

Presently, there is no consensus on why stance duration asymmetry is such a common chronic feature of amputee gait. One possibility is that users preferentially spend more time on their intact limb to minimize time on their prosthesis limb, due to lack of confidence in the prosthesis [6, 18]. Another possibility is that the lack of propulsive power of the prosthesis requires greater impulse from the intact limb [1, 17], which can be achieved by extending the duration of intact leg loading. Consistent with these findings is that stance duration asymmetry is greater for transfemoral amputees compared to transtibial amputees [6]. However, asymmetry has also been shown to decrease with walking speed [1, 6], which suggests that lack of confidence and/or ankle power cannot be the only factors involved. It may also be that users develop locomotor adaptations to optimally accommodate the actions of the prosthesis, as suggested by Maaref et al. [16], but there is presently no mechanistic hypothesis by which to explore this question.

Given the high cost of using a transfemoral prosthesis in terms of energy expenditure [7–9] and fall risk [10, 19], a better understanding of the mechanisms underlying stepping asymmetry is required. Such knowledge could inform designers of above-knee prostheses as well as provide clinicians with a framework for addressing gait asymmetry when training clients to use above-knee prostheses. Our objective in this case study was to develop a mechanistic hypothesis linking compensatory biomechanics and stepping asymmetry in transfemoral amputees.

## 2. Methods

### 2.1. Human Subjects

The study was approved by the University of New Brunswick (Fredericton), Research Ethics Board (REB), and all participants gave their informed consent prior to participation in the study. Participants were included if they were in good physical health and between 19 and 55 years of age. Amputee participants were included if they had a unilateral amputation above or through the knee (>1 yr ago) and normally use a transfemoral prosthesis for daily activity. Participants were excluded for any medical or chronic condition effecting gait or contraindicating moderate physical activity, and any recent injuries requiring treatment (<6 mo) or surgeries (<1 yr) involving the lower extremities and back.

Participants were recruited through the local university community and regional prosthetic clinic. Fifteen limbed adults (7 male, 8 female) and two adult males with transfemoral (through-knee) amputation volunteered to participate in the study.

Both amputee participants lost their lower leg from trauma (>5 years prior to enrolling in this study) that resulted in surgical through-knee disarticulation whereby the distal femur was preserved and the patella-quadriceps complex wrapped distally and sutured to the biceps femoris. Both amputee participants used their currently fitted prosthesis in the study and were recruited through the same local clinic. One participant used a microprocessor-controlled knee (C-Leg® X2 microprocessor knee and Axiton foot from Otto Bock Inc., Duderstadt, Germany), and the other used a hydraulic passive-mechanical controlled knee (Mauch Knee and XC foot from Ossür Inc., Reykjavik, Iceland). Other than the type of prosthesis used, the two amputees' residual limb, socket liners, and clinical management history were similar.

### 2.2. Experimental Procedures

#### 2.2.1. Gait Analysis

**Figure 1:**
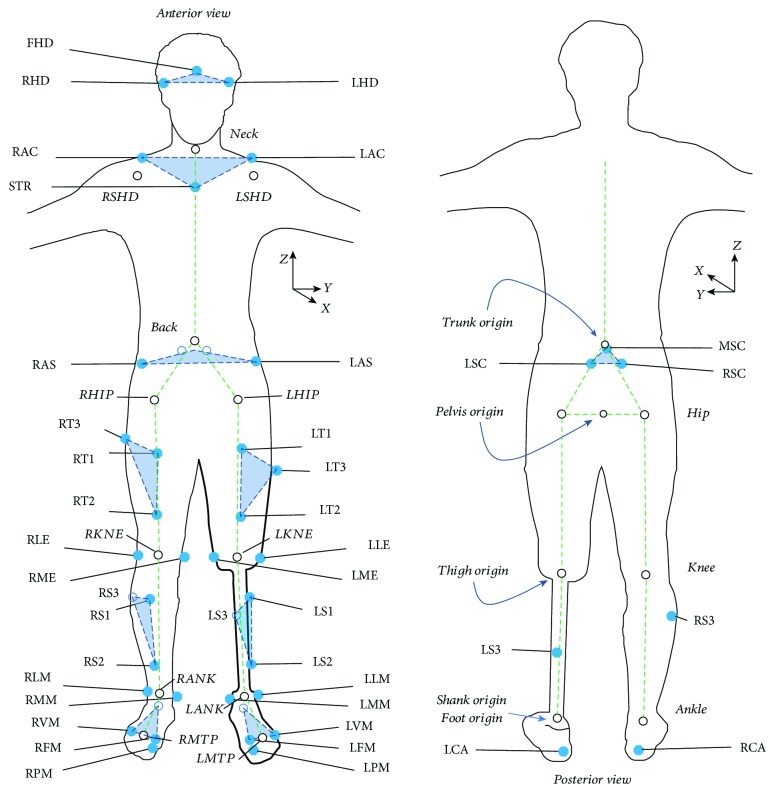
Positioning of the thirty-nine markers used for tracking the musculoskeletal system during movement, which includes triad clusters on each segment plus anatomical markers required to define joint centres and the segment-embedded coordinate system (origins shown, right hand coordinate system), where *x* is anterior pointing, *y* is lateral pointing, and *z* is vertical pointing.

Motion analysis data was collected at the Andrew and Marjorie McCain Human Performance Laboratory (HPL) at the University of New Brunswick. The HPL is equipped with a twelve-camera Vicon T160 (Oxford Metrics, UK) motion tracking system and six Kistler force plates (Kistler Instruments, Winterthur, Switzerland) arranged in a 2 × 3 matrix embedded in the floor. Thirty-nine markers were placed on limbs and torso as shown in Figure 1. All markers (14 mm) were attached to participants' skin (or prosthesis surface) using a double-sided tape, with the exception of the sacral cluster that was a rigid plate with three markers. For amputee participants, markers on the socket, shank, and foot components were attached in similar “anatomical” locations as on the intact limb as indicated in Figure 1 (see also Supplementary Table S1 for marker details).

The experimental protocol began with two sequential 2 s static calibration trials where the participant was asked to stand perfectly still. Participants then completed three constrained chair rise trials [20]. The static standing and chair rise trials were used to generate the body segment model, as described below. Participants were then asked to walk in a straight line through the viewing volume at three different speeds in the following order.

1. Normal (preferred) speed: the subject was instructed to walk at their preferred comfortable pace
2. Fast speed: the subject was instructed to walk as fast as they can without breaking into a jog
3. Slow gait: the subject was instructed to walk as though they were in a slowly moving line

Participants performed at least three repetitions of each gait speed. Trials were repeated (up to six trials) if poor foot strikes were observed, such as neither foot cleanly striking a force plate or two feet on the same plate at the same time. Participants rested 30 s between similar speed conditions and at least 60 s between different speed conditions.

#### 2.2.2. Body Segment Model

As shown in Figure 1, triad clusters were used to track segments and anatomical markers were used to reference joint axes of rotation, for a total of thirty-nine markers (see Table S1 for details). First, each participant's static trial was used to build a subject-specific 6-degree of freedom (6-dof) model of the participant, as described elsewhere [21]. The chair rise trials were used to compute the embedded knee joint flexion/extension axis of rotation, using the SARA algorithm [22]. Hip centres were computed from anatomical scaling as previously described [23]. Anatomical reference frames and inertial properties were taken from Dumas et al. [24].

The resulting 6-dof subject-specific model was then applied to each gait trial of the subject, producing 3D kinematics of left and right foot, shank, thigh, and pelvis. Force plate data were then used with the kinematic data and anatomical (and body segment inertial) data to compute the 3D net joint moments at the ankle, knee, and hip.

The same inverse kinematic and dynamic model was applied to the amputee's prosthetic limb, except the inertial properties of the socket, shank, and foot components were derived from CAD approximations of the user's prosthesis components and the known (measured) mass of each participant's prosthesis. The following adjustments were made to the anatomical model.

The mass of each amputee's residual thigh was first estimated from Dumas' scaling factors and adjusted for atrophy of the residual thigh. Jaegers et al. [25] used MRI to quantify atrophy in residual thigh and found that it could be reduced as much as 30%. Based on clinical judgment, a value of 20% was used. The socket and adjusted residual thigh centres of mass, masses, and inertia tensors were then combined to model the thigh-socket as a rigid link. Knee centres were determined as for limbed participants, using chair rise trials and SARA algorithm [22] for locating the segment-embedded axis of rotation. Although neither amputee's prosthesis had a sagittal plane rotational degree of freedom at the “ankle,” natural deflection of their foot prosthesis allowed for the measurement of an angular displacement and moment of their prosthetic “ankle” (coupling between shaft and foot components), as commonly done in prosthesis gait studies [26]. As such, the intact limb and prosthetic limb are treated by the model in the same way.

#### 2.2.3. Time Normalization

During processing of each subject's trials, custom-written algorithms scanned the foot marker and force plate data to precisely register the stride event frames (*HS-TO-HS*: heel strike–toe off–heel strike) for the left and/or right side. Kinematic and kinetic data were then cycled (using a cubic polynomial spline function, with increment of 1% cycle) between successive heel strikes of the ipsilateral limb for each registered stride. Data for the contralateral limb was also cycled to the ipsilateral *iHS*-*iTO*-*iHS* events to enable analysis of the step-to-step transition (double support phase, *cHS*-*iTO*). By this designation, the ipsilateral limb contacts the floor first (leading limb), followed by the contralateral limb (trailing limb), i.e., *iHS*-*cHS*-*iTO*-*iHS-.*

The 2 × 3 arrangement of force plates enabled us to capture *HS-TO-HS* events for successive strides of both limbs, and most gait trials for control subjects and amputees captured three strides. This produced three sequential (right-left-right or left-right-left) foot step/contacts on three separate plates, thus providing two sequential double support phases: one for the intact side and one for the prosthesis side, as the leading limb.

#### 2.2.4. Data Reduction for Repeated Trials

Even though healthy control subjects can exhibit some gait asymmetry [27], evidence suggests this is small relative to asymmetries observed in users of prostheses [4]. Therefore, for controls, ipsilateral and contralateral cycled data were pooled for left and/or right sides when averaging repeated trials, and then means were taken across the subjects to arrive at sample means and standard deviation boundaries, for each variable in the analysis, and for each gait speed category.

The same approach was used for amputee participants except that left and right sides were not averaged, but rather were assigned to an “intact” and “prosthesis” side. Because this was a case study with *N* = 2, the amputee participants' data were not averaged across subjects.

### 2.3. Biomechanical Analysis

#### 2.3.1. Gait Parameters

Gait parameters included stride parameters and phase parameters. Stride parameters consisted of stride time, the time in seconds (s) elapsed between successive heel strikes of the limb; stride length, the distance in metres (m) between the foot “centre” (defined here as the average of the heel and two metatarsal markers) during their respective (and sequential) mid-stance portion of gait; and stride velocity (m/s), calculated from the stride distance divided by stride time.

Phase parameters consisted of stance phase duration, calculated as the time between *iHS* and *iTO* of the ipsilateral limb, divided by stride time and multiplied by 100, and double support duration was calculated from the time between contralateral limb *cHS* preceding ipsilateral limb *iTO*, divided by stride time and multiplied by 100.

Stride parameters were used to quantify if, and how, the amputees modified their gait speed symmetry. Phase parameters were used to quantify if, and how, the amputees modified the relative timing of stride events (heel strikes and toe off) of the intact and prosthesis side. Gait parameters for slow, normal, and fast speed walking were compared between amputees and control subjects, using single-sample *t*-tests (*α* = 0.01).

#### 2.3.2. External Work on the Body Centre of Mass

Using the approach described by Donelan et al. [28], external work on the CoM was first estimated using ground reaction forces and CoM velocity to estimate the work rate of each limb on the CoM. However, rather than examine the total energy as others have done [14, 17, 29], we separated the interlimb work rate into kinetic and potential components. This was done by first computing the total external work rate (*P*_Ext_) in the sagittal plane for each limb:

(1) $$\begin{array}{c} {P_{\text{Ext}}}^{\text{R}}=F_{x}^{\text{R}}\cdot v_{\text{COM}x}+F_{y}^{\text{R}}\cdot v_{\text{COM}y},\\ {P_{\text{Ext}}}^{\text{L}}=F_{x}^{\text{L}}\cdot v_{\text{COM}x}+F_{y}^{\text{L}}\cdot v_{\text{COM}y}, \end{array}$$

where the R and L superscripts represent right and left limbs, *F*_*y*_ is the vertical ground force and *F*_*x*_ is the anterior-posterior ground force, and CoM velocities are given by *v*_COM_*y*__ and *v*_COM_*x*__ (from the biomechanical model). From here on, we neglect the mediolateral terms in computing the external work, since the internal work methods (below) are limited to the sagittal plane. The kinetic “impulse” work rate of each limb on the CoM was then found from

(2) $$\begin{array}{c} P_{\text{Ext}}^{\text{Imp},\text{R}}={F_{x}}^{\text{R}}\cdot v_{\text{COM}x}+\left({F_{y}}^{\text{R}}-c^{\text{R}*}m^{*}g\right)\cdot v_{\text{COM}y},\\ P_{\text{Ext}}^{\text{Imp},\text{L}}={F_{x}}^{\text{L}}\cdot v_{\text{COM}x}+\left({F_{y}}^{\text{L}}-c^{\text{L}*}m^{*}g\right)\cdot v_{\text{COM}y}, \end{array}$$

where *m* is the total body mass and *g* is the acceleration of gravity (9.81 m/s^2^), and where *c* is the instantaneous proportion of body weight being supported by the limb, or

(3) $$\begin{array}{c} c^{\text{R}}=\frac{{F_{y}}^{\text{R}}}{\left({F_{y}}^{\text{R}}+{F_{y}}^{\text{L}}\right)},\;c^{\text{L}}=1-c^{\text{R}}. \end{array}$$

Finally, the work rate of the limb to overcome gravity of the CoM is found from

(4) $$\begin{array}{c} P_{\text{Ext}}^{\text{Grav},\text{R}}={P_{\text{Ext}}}^{\text{R}}-P_{\text{Ext}}^{\text{Imp},\text{R}}.\\ P_{Ext}^{Grav,L}={P_{Ext}}^{L}-P_{Ext}^{Imp,L} \end{array}$$

Work done by each limb was then computed by integrating the work rate (power) over a specified time interval.

#### 2.3.3. Internal Work of the Leg (and Prosthesis) Joints

Joint net power and mechanical energy flow were calculated as previously described [30] for the ankle, knee, and hip in the sagittal plane, by expressing the net joint power as the sum of the adjacent (distal *d* and proximal *p*) segmental powers at the joint *j*

(5) $$\begin{array}{c} P_{j}=P_{d,j}+P_{p,j}=\tau_{j}\left(\omega_{p}-\omega_{d}\right)={\tau_{j}}^{*}\omega_{j}, \end{array}$$

where the sign of the net power (positive = power generation; negative = power dissipation) dictates whether the joint's muscle action is concentric (power generation) or eccentric (power dissipation). Joint powers were computed about all three axes, but only the sagittal plane data were used in this study. The internal mechanical work of the joints was found from integrating the joint power curve over a specified time interval.

#### 2.3.4. Analysis of the Double Support Phase

The gait cycle phase of interest for this study was the double support phase. During this phase, the step-to-step transfer of forward momentum occurs [31]. This is obviously a critical phase of the gait cycle and is known to be asymmetric in amputees due to the deficiencies in the prosthesis, primarily the weak “push-off” of the ankle/foot component [17]. Amputees' trials were analyzed for two cases (for each gait speed).

**Case 1 .**

Intact side is the “push-off” limb and prosthesis side is the “colliding” limb.

**Case 2 .**

Prosthesis side is the “push-off” limb and intact side as the “colliding” limb.

Of primary interest was the positive and negative external and internal work done by the push-off and colliding limbs during the double support phase.

External work was computed by integrating positive and negative regions of the CoM work rate curves (impulse and gravity). Internal joint work was computed for the positive and negative regions of the joint power curves. External work on the CoM and internal work of joints for amputees was compared to data for the control subjects for the Case 1 and Case 2 trials of slow, normal, and fast speed walking, using single-sample *t*-tests (*α* = 0.01).

### 2.4. Statistical Analysis

For the purpose of this case analysis for developing a hypothesis, we performed mostly descriptive statistics (means and standard deviations), but we also performed quantitative single-subject comparisons between amputees and control subjects for the gait parameters, external CoM work, and internal joint work. A common approach for single-subject comparisons is establishing a threshold for a meaningful change, such as 2 standard deviations from the reference group mean [32, 33]. We used a similar approach except that the threshold was the confidence interval (CI) on the mean of the reference group modelled as a *t*-distribution (appropriate for small samples) with *α* = 0.01, using a custom algorithm written in Matlab (v.R2017b, The MathWorks, Natick, MA). As such, it is similar to conducting a single-sample *t*-test. Although this does not provide inferences to the population of transfemoral amputees, it does provide a way to place confidence on the case-wise identification of compensatory stepping patterns and joint kinetics.

## 3. Results

Participant characteristics are summarized in Table 1. Of the fifteen control subjects, all but one participant had a complete set of slow, normal, and fast speed trials. Therefore, the control subject data was generated from the fourteen participants with complete sets of data. Both amputees also had a complete set of slow, normal, and fast walking trials for both their intact and prosthesis sides.

### 3.1. Gait Parameters

#### 3.1.1. Stride Parameters

Very little asymmetry was found for the stride parameters. As shown in Table 2, there were only minor differences between amputee participants and control subjects for stride length. Stride time was significantly longer (*p* < .01) for the Mauch user's preferred and fast speed gait, and as a result their gait speed was slower than controls (*p* < .01). The C-Leg user's preferred gait speed was slightly faster than control subjects. Importantly, however, the differences relative to control subjects were consistent for both amputees' intact and prosthesis sides, indicating that stride parameters were well matched between intact and prostheses sides or were symmetric.

#### 3.1.2. Phase Parameters

The most striking asymmetry (intact versus prosthesis side) was observed for stance duration, which was longer for the intact limb compared to the prosthesis limb, for both amputees at all three gait speeds. Double support time was slightly asymmetric, but not consistently so; the amputee with the C-Leg had a shorter double support time for their prosthesis limb compared to their intact limb, while the opposite was true for the amputee with the Mauch prosthesis.

In comparison to controls, significant differences were observed in stance duration and double support duration for both amputees. For the amputee with the C-Leg prosthesis, only stance duration of their intact side was significantly longer compared to controls (*p* < .01). This subject's prosthesis side had normal stance phase duration at all three walking speeds. For the amputee with the Mauch prosthesis, the biggest differences were seen in the intact side, but the prosthesis side also had slightly longer stance duration for slow and normal speed walking (both were significant at *p* < .01). Double support time was significantly longer (*p* < .01) for both amputees intact and prostheses sides compared to control subjects.

### 3.2. External CoM and Internal Joint Work

#### 3.2.1. Control Subjects

**Figure 2:**
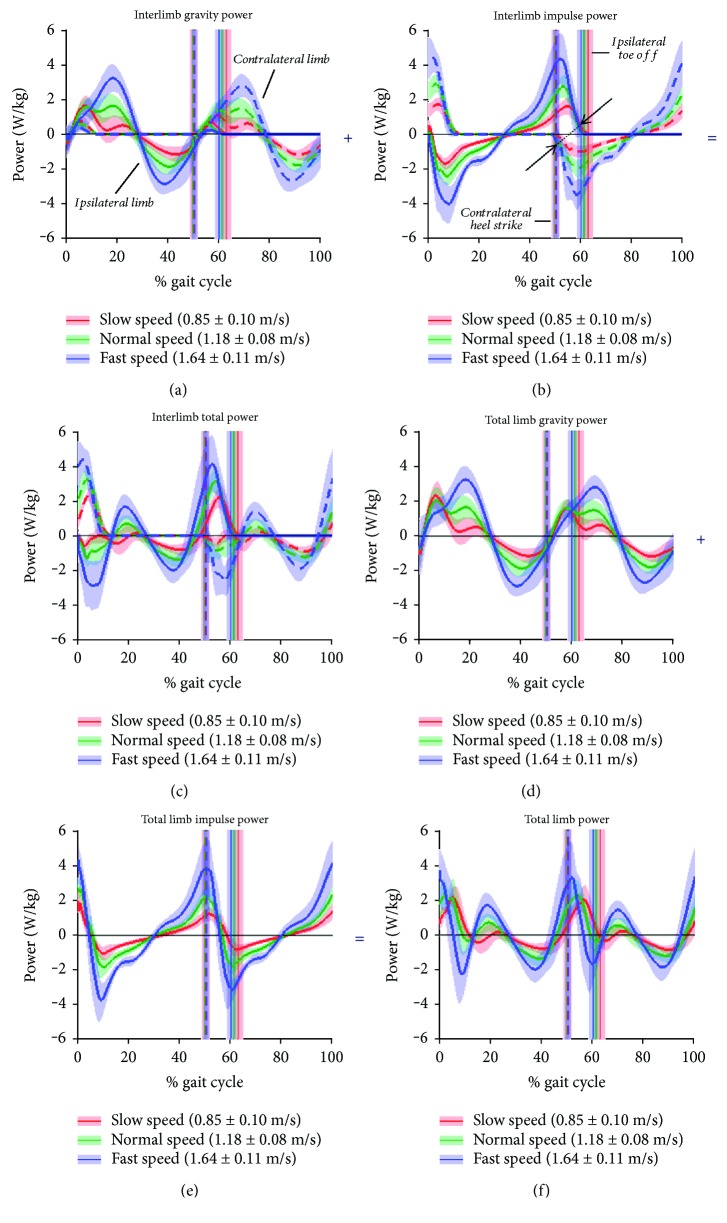
External work on the body CoM during the gait cycle. Data are shown for the ipsilateral limb (solid line) and corresponding contralateral limb (dashed line), for slow (red), normal (green), and fast (blue) speed walking, of nonamputee control subjects. (a, b, c) Work rate of ipsilateral and contralateral limbs to overcome gravity (a) and inertia (b) and the total work rate of each limb (c). (d, e, f) The sum of ipsilateral and contralateral limbs, representing the total work rate of the legs to overcome gravity (d), inertia (e), and total work rate (f). Solid lines represent means across *N* = 14 controls, and shaded boundaries represent ±1 standard deviation from the mean at each % cycle. Vertical solid lines represent toe-off time of the ipsilateral limb, and the dashed vertical lines represent heel strike of the contralateral limb. Shaded boundaries represent ±1 standard deviation in event time.

Figure 2 shows the external work rate on the CoM by the ipsilateral (solid line) and contralateral (dashed line) limbs at slow, normal, and fast walking speed, for the control subjects. The double support period of gait is bracketed by contralateral heel strike (*cHS*) and ipsilateral toe-off (*iTO*) shown by vertical dashed lines. The horizontal axis is time normalized to the 0-100% cycle of the ipsilateral limb, and therefore, the corresponding contralateral limb is also expressed in ipsilateral cycle time. Work rate profiles and magnitudes were similar to other studies of healthy gait [8, 28].

The work rate of each limb to overcome gravity (Figure 2(a)), when summed (Figure 2(d)), shows the smooth transition between limbs for body weight support. Of particular interest in this study was the impulse work rate of each limb (Figure 2(b)) during the double support phase of gait. Note that the timing of the ipsilateral and contralateral “impulse power” on the CoM (Figure 2(b), shown by the arrows) is such that the energy gain from the ipsilateral push-off event is balanced by the contralateral collision event, which result in a smooth transference of propulsive energy (Figure 2(e)).

**Figure 3:**
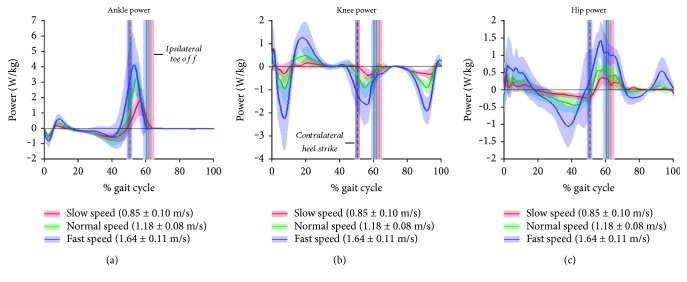
Internal work of the leg joints during the gait cycle. Data are shown for slow (red), normal (green), and fast (blue) speed walking, of nonamputee control subjects. (a, b, c) Work rate of ankle (a), knee (b), and hip (c). Solid lines represent means across *N* = 14 controls, and shaded boundaries represent ±1 standard deviation from the mean at each % cycle. Vertical solid lines represent toe-off time of the ipsilateral limb, and the dashed vertical lines represent heel strike of the contralateral limb. Shaded boundaries represent ±1 standard deviation in event time.

Figure 3 shows ankle, knee, and hip joint power curves for control subjects at slow, normal, and fast walking speeds. Magnitudes were similar to other studies of healthy adult gait [34]. In these plots, the contralateral limb power curves are excluded for clarity. As above, the double support period of gait is bracketed by contralateral heel strike (*cHS*) and ipsilateral toe-off (*iTO*). Joint power profiles behaved as expected for healthy control subjects, having a relatively invariant gait cycle pattern that scales proportionally to walking speed [34]. Plots showing joint angles, moments, and joint power for the full 0-100% cycle, for slow, normal, and fast walking, are shown in Supplementary Figure S2 .

#### 3.2.2. Amputees

Table 3 shows positive and negative external work at slow, normal, and fast speed for control subjects and the two amputees' intact limb and prosthesis limb.  Table 4 shows, in a similar arrangement, the positive and negative internal joint work for the control subjects and two amputees. Single-sample *t*-test results are shown using symbols, where † = significantly lower than control subjects and ‡ = significantly higher than control subjects with an alpha level of 0.01.

External work results in Table 3 illustrate that compared to controls, both amputees did significantly less positive and negative work on the CoM with their prosthesis limb (*p* < .01) and in some cases with their intact limb, particularly for the kinetic impulse work. Internal joint work in Table 4 shows that, with only minor exceptions, amputees did less work than control subjects with their prosthetic ankle and knee and more work with the hip of their prosthesis side (*p* < .01). For amputees' intact limb, there was no difference at the ankle, but amputees did significantly more work than did control subjects with knee and hip of their intact side (*p* < .01).

**Figure 4:**
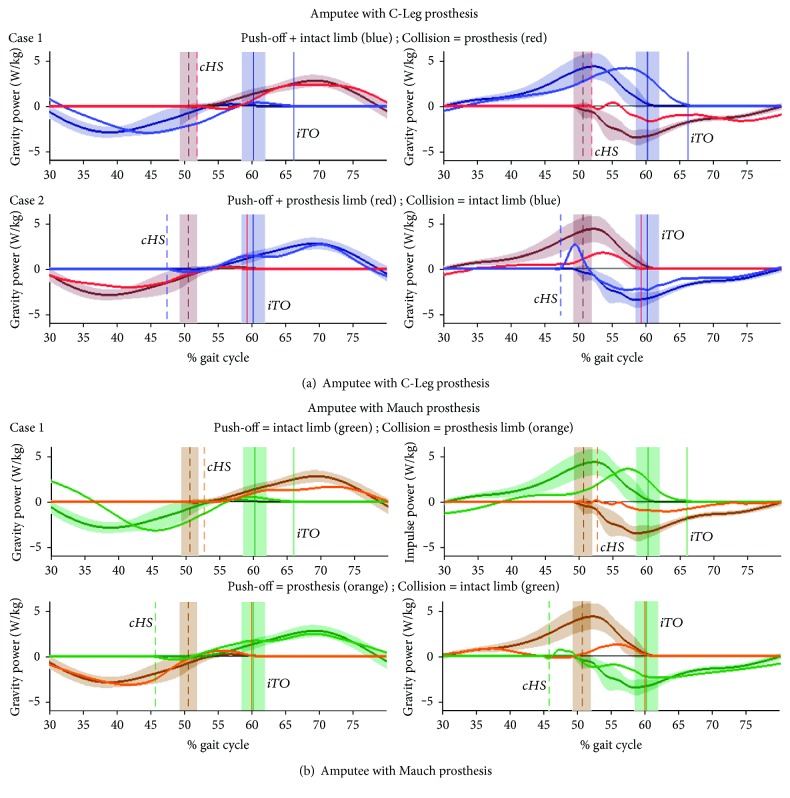
External work on the body CoM during the double support phase of the gait cycle. Data are shown for amputee with C-Leg prosthesis (a) and amputee with Mauch prosthesis (b). The first column of plots shows gravity work rate (power) on centre of mass (CoM), and the 2nd column shows impulse work rate (power) on CoM. For each amputee, the first row shows Case 1 where the intact limb is the push-off limb (blue) and Case 2 where the push-off limb is the prosthesis (red line). The mean for control subjects (*N* = 14) is shown by dark solid lines with shaded boundaries that represent ±1 standard deviation from the mean at each % cycle. Vertical solid lines represent toe-off time of the ipsilateral limb, and the dashed vertical lines represent heel strike of the contralateral limb, and the shaded boundaries represent ±1 standard deviation in event time. The horizontal axis shows the 30-80% gait cycle.

**Figure 5:**
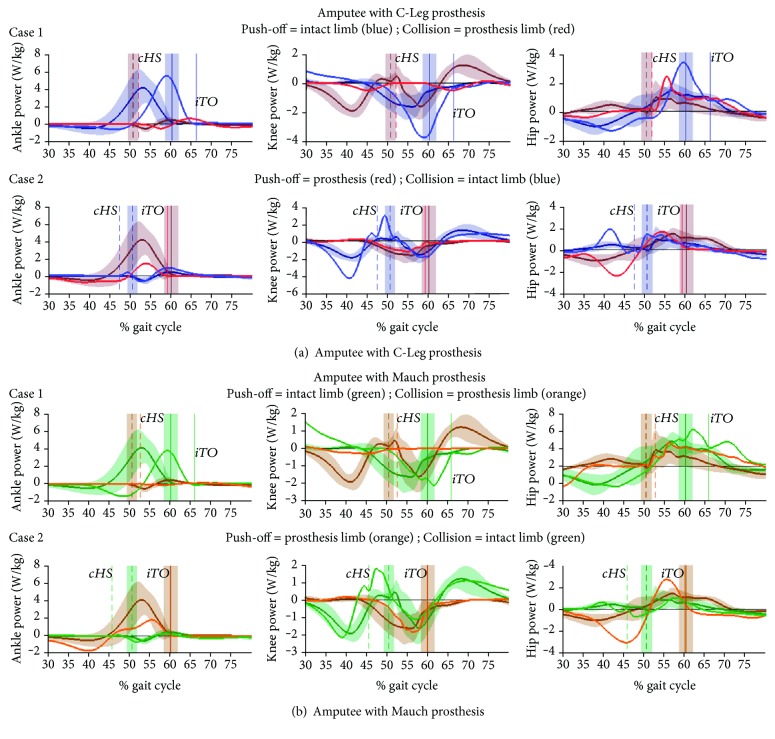
Internal joint work rate (power) during the double support phase of the gait cycle. Data are shown for amputee with C-Leg prosthesis (a) and amputee with Mauch prosthesis (b). The first column of plots shows ankle power, the 2^nd^ column shows knee power, and the 3^rd^ column shows hip power. For each amputee, the first row shows Case 1 where the intact limb is the push-off limb (blue) and Case 2 where the push-off limb is the prosthesis (red line). The mean for control subjects (*N* = 14) is shown by dark solid lines with shaded boundaries that represent ±1 standard deviation from the mean at each % cycle. Vertical solid lines represent toe-off time of the ipsilateral limb, and the dashed vertical lines represent heel strike of the contralateral limb, and the shaded boundaries represent ±1 standard deviation in event time. The horizontal axis shows the 30-80% gait cycle.

Figure 4 shows the gravity and impulse work rate on the CoM for the two amputee subjects, against the means for control subjects' ipsilateral and contralateral limbs with standard deviation boundaries, at their fast walking speed. Joint (ankle, knee, and hip) power plots for amputees are similarly arranged in Figure 5. The time scale of plots in Figures 4 and 5 were set to 30-80% cycle in order to more clearly visualize the double support phase. Plots showing external work rate and internal joint power for the full 0-100% cycle, for slow, normal, and fast walking, are shown in Supplementary Figure S3 .

Results for the amputee with the C-Leg prosthesis are shown in Figure 4(a) and Figure 5(a) (blue = intact, red = prosthesis), and results for the amputee with the Mauch hydraulic knee prosthesis are shown in Figure 4(b) and Figure 5(b) (green = intact, orange = prosthesis). Ipsilateral toe-off (*iTO*) events are shown by vertical solid lines, and contralateral heel strike (*cHS*) events are shown by vertical dashed lines (and with s.d. boundaries for control subjects).

## 4. Discussion

Whether lack of confidence in the prosthesis causes users to spend more time on their intact limb during stance phase of gait, or users extend stance of the intact limb to increase impulse generation [6], users of transfemoral prostheses must adapt to both the actions and the deficiencies of the prosthesis [16]. Although increased internal work [7, 13, 35] is suspected as playing a role in compensating for lack of external work on the CoM by the prosthesis [8, 17], an understanding of how this compensation relates to stance duration asymmetry is lacking for transfemoral amputees. The primary purpose of this study was to develop a mechanistic hypothesis linking compensatory biomechanics and stepping asymmetry in TF amputees.

### 4.1. Stepping Asymmetry

Clearly evident for both amputees' Case 1 in both Figures 4 and 5 is the delayed *iTO* event for the intact limb, occurring later in the gait cycle, by more than 5% and well outside the shaded boundary region on the *iTO* event of control subjects. Also notable was that the *cHS* event for the colliding prosthesis limb, in intact limb “cycle time,” was also delayed compared to controls. Although a smaller departure, for one amputee (Mauch) the *cHS* event fell outside the shaded boundary on the *cHS* region for controls, and for the other (C-Leg) it was located at the edge of the shaded region.

For Case 2, the *iTO* event of the push-off prosthesis limb for both amputees was slightly earlier compared to controls, but within the control *iTO* boundary. The *cHS* event of the amputees' colliding intact limb, in prosthesis “cycle time,” occurred approximately 3-5% earlier in the cycle, consistent with a faster swing phase to compensate for the longer stance duration.

These event departures reflect that the primary consequence of motor adaptations to the prosthesis have resulted in a stepping asymmetry characterized by increased stance duration (and reduced swing time) of the intact side of amputee participants, while maintaining (relative to controls) normal phase parameters of the prosthesis side.

### 4.2. Compensatory Biomechanics

#### 4.2.1. External Work on CoM

Plots for the C-Leg user (Figure 4(a)) and Mauch user (Figure 4(b)) identify how the energy transfer from the legs to and from the CoM is able to accommodate the asymmetry in stance duration. The first column of plots showing the interlimb work rate of gravity on the CoM reveals a relatively normal pattern for both amputees when their prosthetic limb was the push-off limb (Case 2). When the push-off limb was the intact limb, however, the work rate of gravity was delayed for the intact side (Case 1). This effect was present for both amputees but more noticeable for the Mauch Knee user.

Most revealing were the observed differences between amputees and control subjects in the pattern of interlimb impulse work rate on the CoM. These characteristics were consistent for both the C-Leg and Mauch user at all three gait speeds (see also Figure S3 ).

For Case 1, when the intact limb (blue line) was the push-off limb, the lengthened stance (delayed *iTO*) appeared to accommodate the slow development of negative work on the colliding prosthetic limb (red line). Indeed, the negative work rate of the prosthesis side following *cHS* was considerably lower than for controls, but nevertheless the transfer of energy from the intact to prosthesis side maintained its principle form.

For Case 2, when the prosthesis limb (red line) was the push-off limb, the impulse power generated by the prosthesis side at push-off was, as expected, significantly lower than for controls, although the *iTO* event for the prosthesis limb was the same as for control subjects. For the colliding intact limb (blue line), the earlier *cHS* event appeared to enable a brief positive power region that was not present for controls. In other words, the colliding intact limb was carrying out a positive power task prior to taking on its role to accept energy from the transferring push-off limb. This appears to compensate in part for the reduced positive work of the push-off limb, by accelerating the CoM with the intact leg just after heel strike, which is timed earlier to allow for the “normal” transfer of weight support.

For the two amputees we observed, their prosthetic limb did little to contribute to impulse work during push-off and collision. The weak collision of the prosthesis limb was compensated by extending stance duration of the intact limb. Then, during the weak push-off of the prosthesis limb, the intact side compensated by adding positive power prior to push-off of the prosthesis limb. We now examine the potential sources for these compensations.

#### 4.2.2. Internal Work of Joints

Joint power plots for the C-Leg user (Figure 5(a)) and Mauch user (Figure 5(b)) identify the internal sources that explain the above compensations. For Case 1 (intact limb is push-off limb) of both amputees, the ankle plantar-flexion power burst at push-off (blue line) was the same as for control subjects, just delayed in cycle time. Also delayed was the late stance negative power region of the intact knee (blue line) that followed a significant positive power region in the earlier portion of stance phase, as seen at the lower boundary (30% cycle) of the knee power plots for Case 1. Additionally, the peak positive and negative powers for the hip of the intact limb (blue line) were delayed and had greater peak magnitudes than in control subjects. Power profiles of the colliding prosthesis limb show no effective response at the knee, and possibly higher hip power of the prosthesis limb following heel strike, although this was not consistent for the two amputees.

For Case 2 (prosthesis limb is push-off limb) of both amputees, the timing of the artificial ankle/foot power burst was similar to controls but the magnitude was significantly attenuated. The compensatory function of the intact knee (blue line) of the colliding limb, however, is clearly evident, in particular the spike in positive knee power just following heel strike, when normally the knee would be dissipating power at load acceptance. For the hips, the push-off prosthesis limb (red line) had a significant negative power region between the 40 and 45% cycle that preceded the intact limb's heel strike (*cHS*).

### 4.3. A Mechanistic Hypothesis for Stepping Asymmetry

Overall, Figures 4 and 5 demonstrate the similarity in asymmetric stepping patterns of the intact and prosthetic limbs of the two amputees. Although the two amputees used very different prostheses (both knee and foot components), they both appeared to adapt to their prosthesis in the same way. Waveforms for normal speed and slow speed walking showed the same asymmetry patterns (also see Figure S3), indicating that the stepping asymmetry observed was not a function of speed.

These findings suggest that transfemoral amputees modify both heel strike time (in prosthesis side cycle time) and toe-off time (in intact limb cycle time) to enable the stance phase to be lengthened and the swing phase to be shortened. The shorter swing phase of the intact limb was timed to collide earlier relative to the prosthesis limbs' cycle to enable a transfer of positive power to the CoM prior to the prosthesis side push-off, while extending intact limb stance duration to compensate for collision work deficiency of the prosthesis. The data suggest that the intact knee joint plays a pivotal role in this process.

While the hip of the intact limb was clearly compensating for power generation at push-off, the role of the hip earlier in the gait cycle was not as clear from the data. Of particular interest though was the substantial negative work done by the hip of the prosthesis limb in late stance. This characteristic has been reported for amputees [13, 35, 36] and has also been observed in seniors with disability [37] and may be a mechanism for transferring energy to the upper body [38], which for the amputee would otherwise be wasted by the prosthesis' inability to return that energy.

### 4.4. Limitations

There are several notable limitations of the study. Most significant was having only two participants with limb amputation. Furthermore, the degree of stepping asymmetry was similar but not identical for the two amputees, which is probably related to individual differences and those related to their specific prosthesis. However, in the context of the study's objective, and with the very good agreement with past literature, we feel our conclusions are well supported. Larger studies examining these effects over time, from first fitting to long-term follow-up, will likely be more informative than studies with large N. Nevertheless, these studies will be required to definitively answer the question if neural reorganization is responsible for these adaptations and to what end.

A more significant limitation may be in generalizability of the results to the above-knee amputee population, given that both participants had had through-knee disarticulation amputations, which results in a long residual limb and causes the prosthesis knee axis to be more distal than the intact knee axis. Although this geometric asymmetry could play a role, studies examining residual limb length effects on amputee gait generally show little, if any, difference in the biomechanics of gait for longer versus shorter residual limbs [11, 16]. However, we are not able to analyze this effect with our current data. Future studies should include individuals with different levels of amputation.

Another limitation is how we controlled gait speed. Although there is an argument for using a treadmill to ensure experimental control of gait speed, we opted for the more ecologically realistic condition of over-ground walking. While pace control can still be implemented with over-ground walking (e.g., using a metronome), we instead chose to use a set of verbal instructions (i.e., “walk as if…”) that would be contextually understood for each of the three self-selected speeds. Given that we observed the same adaptations and compensations in both amputees at all three self-selected gait speeds, suggests that using self-selected speeds may be more of a strength than a weakness. Had we controlled speeds artificially, it could be argued that the compensations observed were specific to “non-self-selected” speeds and thus less valuable clinically.

Finally, our model was not complete. Firstly, we neglected any external work due to the force couple on the CoM caused by a translating centre of pressure. Mathematically, this is equivalent to a slipping contact, but its contribution to external work during walking has been traditionally neglected (c.f. [28, 31, 39–41]). Future studies might evaluate the validity of this assumption. We also used crude estimates of the mass of the amputee's residual thigh; sensitivity analyses in future modelling efforts will be required. Also, we did not examine the power flow to and from the upper body. The highly deficient negative work of the prosthesis limb on the CoM suggests that internal work of the musculoskeletal system is managing a more complex behavior at and above the hips that warrants future attention.

## 5. Conclusions

Our study supports the notion that stepping asymmetry in users of artificial limbs is an adaptation to increase functionality and safety of their gait, which has been observed both in gait re-education programs [42] and in model simulations [17]. Despite using very different prostheses, the two amputees demonstrated very symmetric stride characteristics (stride length and speed), and the stance/swing duration of the prosthesis limb was more similar to control subjects than the amputee's intact side. This may reflect that they were > 5 years since starting to use their current prosthesis and thus had “fined-tuned” their gait to maximize symmetry of speed (stride time and distance).

The asymmetry in stance duration was characterized by significant alteration of intact limb heel strike and toe-off events, all the while a near normal stance/swing phase for the prosthesis limb was being achieved. This may be a constraint induced by the advanced control mechanisms of the two devices (the C-Leg and Mauch knees provide both stance and swing phase control), which were intelligent enough to enforce a relatively normal periodicity upon the prosthesis limb (i.e., ~60% stance and ~40% swing).

Although a rationale design feature, the data from the present study and past studies would suggest that this enforcement does not overcome the deficiency of the above-knee prosthesis to do the required positive work during push-off and negative work during collision.

Our data are supported by most, if not all, of the prior studies that show increased concentric energy expenditure of the intact knee in stance phase [35], increased concentric energy expenditure of both the intact and prosthesis side hips [7, 13, 35], and increased negative work of the prosthetic side hip in late stance [13]. However, our analysis goes beyond these studies by identifying the connection between these compensations and the adapted heel strike and toe-off events of the intact limb.

The extended stance duration of the intact limb has been suggested as a strategy to increase the impulse of the intact limb on the CoM [16, 18], which indeed may be a consequence, but our data suggest that the motor program of the intact leg is purposefully delayed to allow two key compensations to occur: (1) a brief period of positive work added by the intact limb following its collision, to supplement the weak push-off of the prosthesis limb, which allows (2) the more robust push-off leg to time its delivery to minimize the influence of the deficient collision work of the prosthesis limb.

Based on the data, we suspect that physical interventions attempting to reestablish “normality” of the intact leg's stance and swing duration, without improvements to the prosthesis, could result in less safe walking. Our data, though limited, suggests that the solution is to focus efforts on better push-off and collision control of the prosthesis.

## Figures and Tables

**Table 1:** Participant characteristics (mean ± standard deviation) for controls (*N* = 14) and two transfemoral amputees.

Subjects	Prosthesis	Age (years)	Height (cm)	Body mass (kg)	Sex
Controls		27 ± 7.5	169 ± 9.2	68.6 ± 12.5	*M* = 6; *F* = 8
Amputee	C-Leg	31	178	75	M
Amputee	Mauch	34	180	63	M

**Table 2:** Gait parameters measured for controls (*N* = 14) and two transfemoral amputees during slow, normal, and fast speed gait, and results of the single sample *t*-test between amputee and sample of control subjects.

	Control subjects			Amputee: C-Leg/Mauch					
	Mean/(SD)			Intact side			Prosthesis side		
	Slow	Norm	Fast	Slow	Norm	Fast	Slow	Norm	Fast
Stride params									
Stride time (s)	1.40 (0.22)	1.07 (0.07)	0.86 (0.09)	1.43	1.02	0.91	1.43	1.02	0.93
				1.48	1.25^‡^	1.03^‡^	1.48	1.23^‡^	1.04^‡^
Stride dist. (m)	1.16 (0.08)	1.26 (0.08)	1.40 (0.13)	1.11	1.29	1.49	1.07^†^	1.32	1.46
				1.16	1.32	1.46	1.13	1.27	1.45
Stride vel. (m/s)	0.85 (0.14)	1.18 (0.11)	1.64 (0.16)	0.78	1.27^‡^	1.63	0.75	1.29^‡^	1.57
				0.78	1.05^†^	1.41^†^	0.77	1.03^†^	1.40^†^
Phase params									
Stance duration (% cycle)	61.5 (1.97)	59.2 (1.19)	57.7 (1.89)	70.6^‡^	65.0^‡^	63.9^‡^	62.8	59.0	57.0
				70.2^‡^	65.6^‡^	63.9^‡^	64.1^‡^	62.6^‡^	57.9
Double support (% cycle)	12.9 (1.86)	11.2 (0.96)	9.57 (1.44)	20.3^‡^	14.8^‡^	14.3^‡^	16.2^‡^	13.1^‡^	11.8^‡^
				17.2^‡^	14.4^‡^	13.2^‡^	18.9^‡^	17.9^‡^	14.2^‡^

^†^Score is significantly lower at *p* < .01; ^‡^score is significantly higher at *p* < .01.

**Table 3:** Interlimb external work on CoM for controls and two transfemoral amputees during the 0-100% gait cycle of slow, normal, and fast speed walking, with results from the single sample *t*-test between amputee and sample of control subjects. Wp = positive work (J/kg); Wn = negative work (J/kg); Wt = total work (J/kg), where Wt = Wp + ∣Wn∣.

Work (J/kg)	Control subjects			Amputee: C-Leg/Mauch					
	Mean/(SD)			Intact side			Prosthesis side		
	Slow	Norm	Fast	Slow	Norm	Fast	Slow	Norm	Fast
Impulse									
Wp	0.295 (0.061)	0.367 (0.078)	0.500 (0.138)	0.254	0.423	0.658^‡^	0.108^†^	0.126^†^	0.234^†^
				0.218^†^	0.254^†^	0.450	0.057^†^	0.087^†^	0.151^†^
Wn	0.307 (0.046)	0.372 (0.074)	0.525 (0.125)	0.105^†^	0.268^†^	0.369^†^	0.168^†^	0.251^†^	0.294^†^
				0.069^†^	0.130^†^	0.572	0.156^†^	0.184^†^	0.134^†^
Wt	0.602 (0.083)	0.739 (0.145)	1.024 (0.245)	0.359^†^	0.691	1.027	0.276^†^	0.377^†^	0.528^†^
				0.287^†^	0.384^†^	1.021	0.213^†^	0.271^†^	0.285^†^
Gravity									
Wp	0.320 (0.062)	0.354 (0.079)	0.442 (0.112)	0.249^†^	0.320	0.402	0.238^†^	0.297	0.348
				0.297	0.315	0.664^‡^	0.264^†^	0.378	0.303^†^
Wn	0.281 (0.055)	0.327 (0.084)	0.402 (0.107)	0.277	0.350	0.492	0.224^†^	0.289	0.289^†^
				0.332^‡^	0.341	0.455	0.261	0.413^‡^	0.466
Wt	0.601 (0.105)	0.681 (0.154)	0.844 (0.210)	0.526	0.670	0.894	0.462^†^	0.585	0.637^†^
				0.629	0.656	1.119^‡^	0.525	0.791	0.769

^†^Significantly lower at *p* < .01; ^‡^Significantly higher at *p* < .01.

**Table 4:** Internal joint work for controls and two transfemoral amputees during the 0-100% gait cycle of slow, normal, and fast speed walking, with results from the single sample *t*-test between amputee and sample of control subjects. Wp = positive work (J/kg); Wn = negative work (J/kg); Wt = total work (J/kg), where Wt = Wp + ∣Wn∣.

Work (J/kg)	Control subjects			Amputee: C-Leg/Mauch					
	Mean/(SD)			Intact side			Prosthesis side		
	Slow	Norm	Fast	Slow	Norm	Fast	Slow	Norm	Fast
Ankle									
Wp	0.212 (0.061)	0.276 (0.083)	0.352 (0.119)	0.193	0.315	0.418	0.035^†^	0.068^†^	0.083^†^
				0.211	0.256	0.286	0.080^†^	0.095^†^	0.194^†^
Wn	0.179 (0.029)	0.154 (0.033)	0.119 (0.071)	0.177	0.154	0.135	0.135^†^	0.146	0.164
				0.186	0.171	0.199^‡^	0.192	0.205^‡^	0.225^‡^
Wt	0.391 (0.047)	0.430 (0.075)	0.471 (0.130)	0.371	0.469	0.554	0.170^†^	0.214^†^	0.247^†^
				0.397	0.428	0.485	0.272^†^	0.299^†^	0.420
Knee									
Wp	0.049 (0.037)	0.089 (0.047)	0.158 (0.076)	0.088^‡^	0.182^‡^	0.191	0.020	0.016^†^	0.015^†^
				0.121^‡^	0.200^‡^	0.363^‡^	0.012^†^	0.013^†^	0.015^†^
Wn	0.139 (0.056)	0.249 (0.079)	0.442 (0.081)	0.327^‡^	0.587^‡^	0.714^‡^	0.093	0.180^†^	0.192^†^
				0.360^‡^	0.371^‡^	0.469	0.115	0.132^†^	0.203^†^
Wt	0.188 (0.087)	0.338 (0.118)	0.600 (0.140)	0.415^‡^	0.769^‡^	0.905^‡^	0.114^†^	0.196^†^	0.208^†^
				0.481^‡^	0.572^‡^	0.832^‡^	0.127	0.145^†^	0.219^†^
Hip									
Wp	0.103 (0.051)	0.144 (0.067)	0.278 (0.095)	0.201^‡^	0.340^‡^	0.466^‡^	0.106	0.215^‡^	0.245
				0.336^‡^	0.345^‡^	0.445^‡^	0.196^‡^	0.230^‡^	0.392^‡^
Wn	0.083 (0.032)	0.120 (0.054)	0.185 (0.064)	0.078	0.145	0.235	0.162^‡^	0.281^‡^	0.307^‡^
				0.048^†^	0.143	0.300^‡^	0.218^‡^	0.343^‡^	0.497^‡^
Wt	0.186 (0.052)	0.264 (0.065)	0.463 (0.076)	0.279^‡^	0.486^‡^	0.700^‡^	0.268^‡^	0.496^‡^	0.552^‡^
				0.384^‡^	0.487^‡^	0.744^‡^	0.414^‡^	0.573^‡^	0.889^‡^

^†^Significantly lower at *p* < .01; ^‡^significantly higher at *p* < .01.

## Data Availability

The data used to support the findings of this study are available from the corresponding author upon request.

## Supplementary Materials

Supplementary 1 S1: motion analysis marker descriptions. Table S1 contains detailed information about body marker locations used for control subjects and amputee participants.

Supplementary 2 S2: joint kinematics and kinetics. Graphs shown in the manuscript are limited to double-support phase (30%-80% gait) of fast gait. For the reader to see the whole gait cycle for all three gait speeds, we include the following supplements for walking trials at slow, preferred, and fast speed, from 0 to 100% gait cycle (heel strike to heel strike). Figure S2.1 contains joint angles and moments for healthy controls with data for two amputees (C-Leg user and Mauch user) superimposed. Figure S2.2 contains joint and segment powers for healthy controls with data for two amputees (C-Leg user and Mauch user) superimposed.

Supplementary 3 S3: external and internal work rate. These graphs show results for the 0-100% gait cycle for the intact limb and for the prosthetic limb, at each of the three speeds, for each of the two amputees. Figure S3.1 contains external work rate (gravity and impulse) for healthy controls with data for two amputees (C-Leg user and Mauch user) superimposed. Figure S3.2 contains internal work rate (ankle, knee, and hip) for healthy controls with data for two amputees (C-Leg user and Mauch user) superimposed.
