# Supplementary material for: Development of a Mechanistic Hypothesis Linking Compensatory Biomechanics and Stepping Asymmetry during Gait of Transfemoral Amputees
Source: Appl Bionics Biomech. 2019 Feb 3;2019:4769242. doi: 10.1155/2019/4769242 (PMC6378070; doi:10.1155/2019/4769242)
Supplement: Supplementary 1 — S1: motion analysis marker descriptions. Table S1 contains detailed information about body marker locations used for control subjects and amputee participants. [file 4769242.f1.pdf]

S1 – Motion analysis marker locations

Table S1. Marker clusters and description.

Marker Cluster	Segment/ Joint	Description
FHD, RHD, LHD	Head	Front of head (forehead) and right and left side of the head (temples)
STR, RAC, LAC	Trunk	Sternum (proximal) and superior aspect of right and left acromion processes
RAS, LAS	Pelvis	Right and left anterior superior iliac spine (ASIS) processes
RSC, LSC, MSC	Pelvis/Sacrum	Right and left posterior superior iliac spine (PSIS) and lumbo-sacral joint (dorsal surface)
RT1, RT2, RT3	Right Thigh	Right thigh upper, lower (anterior) and lateral
LT1, LT2, LT3	Left Thigh	Left thigh upper, lower (anterior) and lateral
RLE, RME	Right Knee	Right lateral and medial epicondyle, distal femur
LLE, LME	Left Knee	Left lateral and medial epicondyle, distal femur
RS1, RS2, RS3	Right Shank	Right shank upper, lower (anterior) and lateral
LS1, LS2, LS3	Left Shank	Left shank upper, lower (anterior) and lateral
RLM, RMM	Right Ankle	Right lateral and medial malleolus, distal tibia
LLM, LMM	Left Ankle	Left lateral and medial malleolus, distal tibia
RCA, RVM, RFM	Right Foot	Right heel (calcaneus), fifth metatarsal head (small toe) and first metatarsal head (great toe)
RPM	Right Toes	Right first phalangeal, great toe tip
LCA, LVM, LFM	Left Foot	Left heel (calcaneus), fifth metatarsal head (small toe) and first metatarsal head (great toe)
LPM	Left Toes	Left first phalangeal, great toe tip
