# Supplementary material for: Development of a Mechanistic Hypothesis Linking Compensatory Biomechanics and Stepping Asymmetry during Gait of Transfemoral Amputees
Source: Appl Bionics Biomech. 2019 Feb 3;2019:4769242. doi: 10.1155/2019/4769242 (PMC6378070; doi:10.1155/2019/4769242)
Supplement: Supplementary 2 — S2: joint kinematics and kinetics. Graphs shown in the manuscript are limited to double-support phase (30%-80% gait) of fast gait. For the reader to see the whole gait cycle for all three gait speeds, we include the following supplements for walking trials at slow, preferred, and fast speed, from 0 to 100% gait cycle (heel strike to heel strike). Figure S2.1 contains joint angles and moments for healthy controls with data for two amputees (C-Leg user and Mauch user) superimposed. Figure S2.2 contains joint and segment powers for healthy controls with data for two amputees (C-Leg user and Mauch user) superimposed. [file 4769242.f2.pdf]

S2 – Sagittal plane joint kinematics and kinetics

S2 Fig 1. Sagittal plane joint angles and moments. A: Slow speed gait; B: Normal (preferred) speed gait; and C: Fast speed gait. Controls are shown with black lines with gray boundaries representing +/- 1 standard deviation (s.d.). Blue lines are for an amputee with a C-Leg micro-controlled prosthesis and green lines are for an amputee with a passive-mechanical Mauch hydraulic knee. For amputees, dash line is prosthesis side and solid line is their intact side. Vertical lines show toe-off time for controls (gray region representing +/-1 s.d.) as well as toe-off of the prosthesis side (dashed vertical line) and intact side (solid vertical line) for both amputees. Top rows of each panel are ankle, knee and hip joint angle, and bottom rows are net joint torques for ankle, knee and hip.

S2 Fig 2. Sagittal plane joint and segment powers for fast speed gait. A: Slow speed gait; B: Normal (preferred) speed gait; and C: Fast speed gait. Controls are shown with black lines with gray boundaries representing +/- 1 standard deviation (s.d.). Blue lines are for an amputee with a C-Leg micro-controlled prosthesis and green lines are for an amputee with a passive-mechanical Mauch hydraulic knee. For amputees, dash line is prosthesis side and solid line is their intact side. Vertical lines show toe-off time for controls (gray region representing +/-1 s.d.) as well as toe-off of the prosthesis side (dashed vertical line) and intact side (solid vertical line) for both amputees. Top rows of each panel are distal segment powers at each joint, middle rows are proximal segment powers at each joint, and the bottom row shows the resulting “net joint power”, at each joint.

Figure S2.1(A)

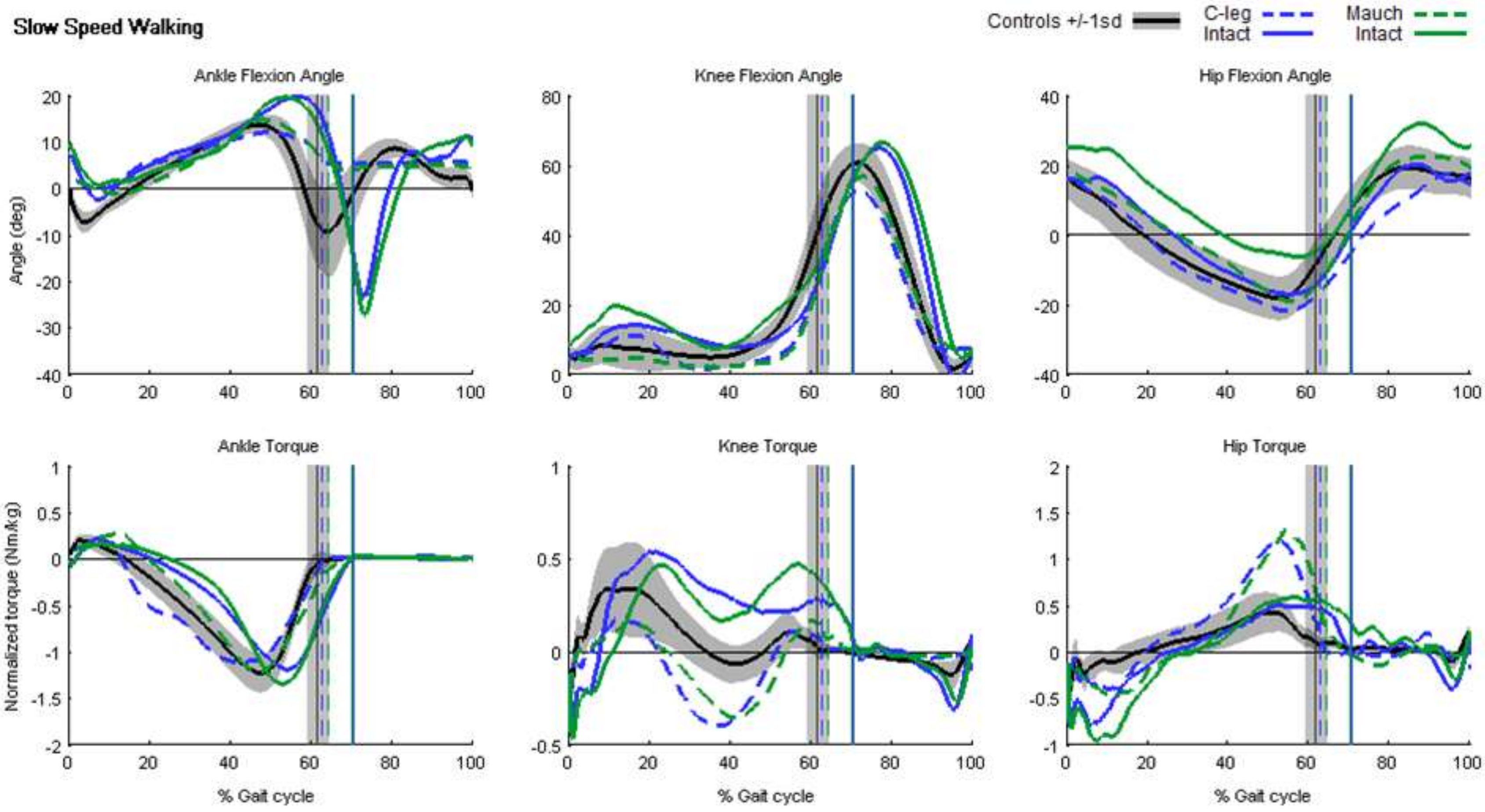
Slow Speed Walking
Controls +/-1sd
C-leg
Intact
Mauch
Intact
Ankle Flexion Angle
Knee Flexion Angle
Hip Flexion Angle
Angle (deg)
Ankle Torque
Knee Torque
Hip Torque
Normalized torque (Nm/kg)
% Gait cycle
% Gait cycle
% Gait cycle

Figure S2.1(B)

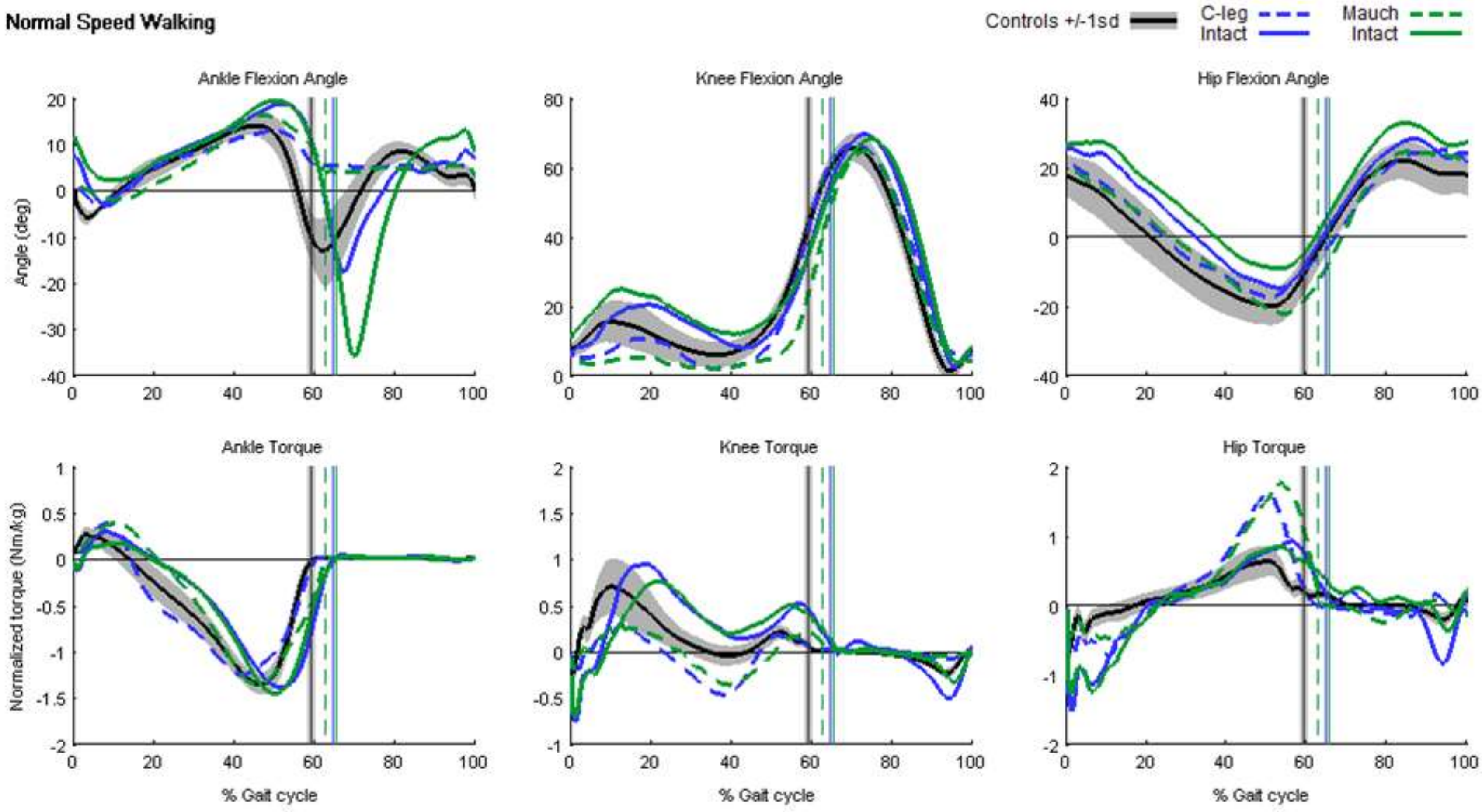
Normal Speed Walking
Controls +/-1sd
C-leg
Intact
Mauch
Intact
Ankle Flexion Angle
Knee Flexion Angle
Hip Flexion Angle
Angle (deg)
Ankle Torque
Knee Torque
Hip Torque
Normalized torque (Nm/kg)
% Gait cycle
% Gait cycle
% Gait cycle

Figure S2.1(C)

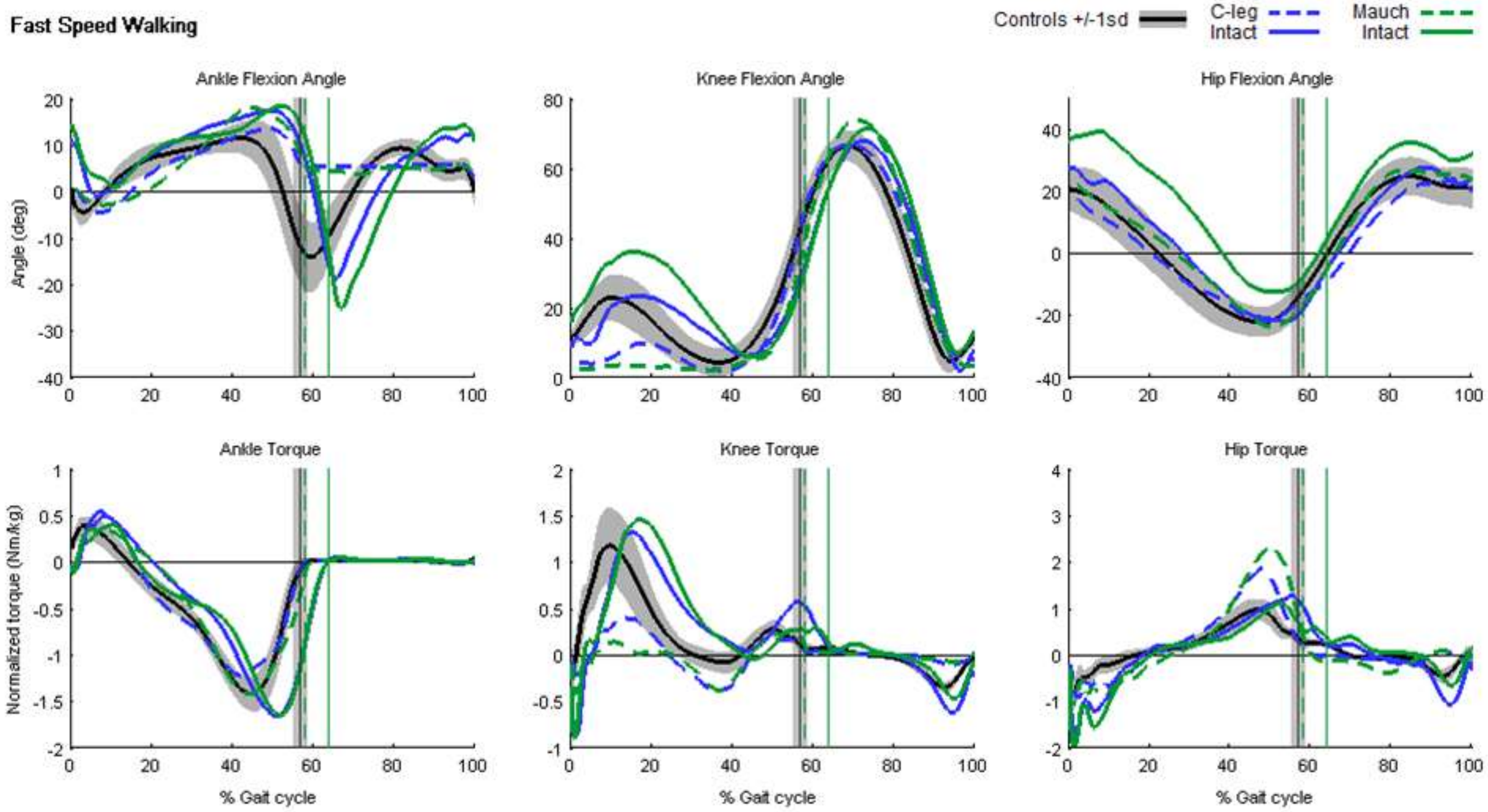

Fast Speed Walking
Controls +/-1sd
C-leg
Intact
Mauch
Intact
Ankle Flexion Angle
Knee Flexion Angle
Hip Flexion Angle
Angle (deg)
Ankle Torque
Knee Torque
Hip Torque
Normalized torque (Nm/kg)
% Gait cycle

Figure S2.2(A)

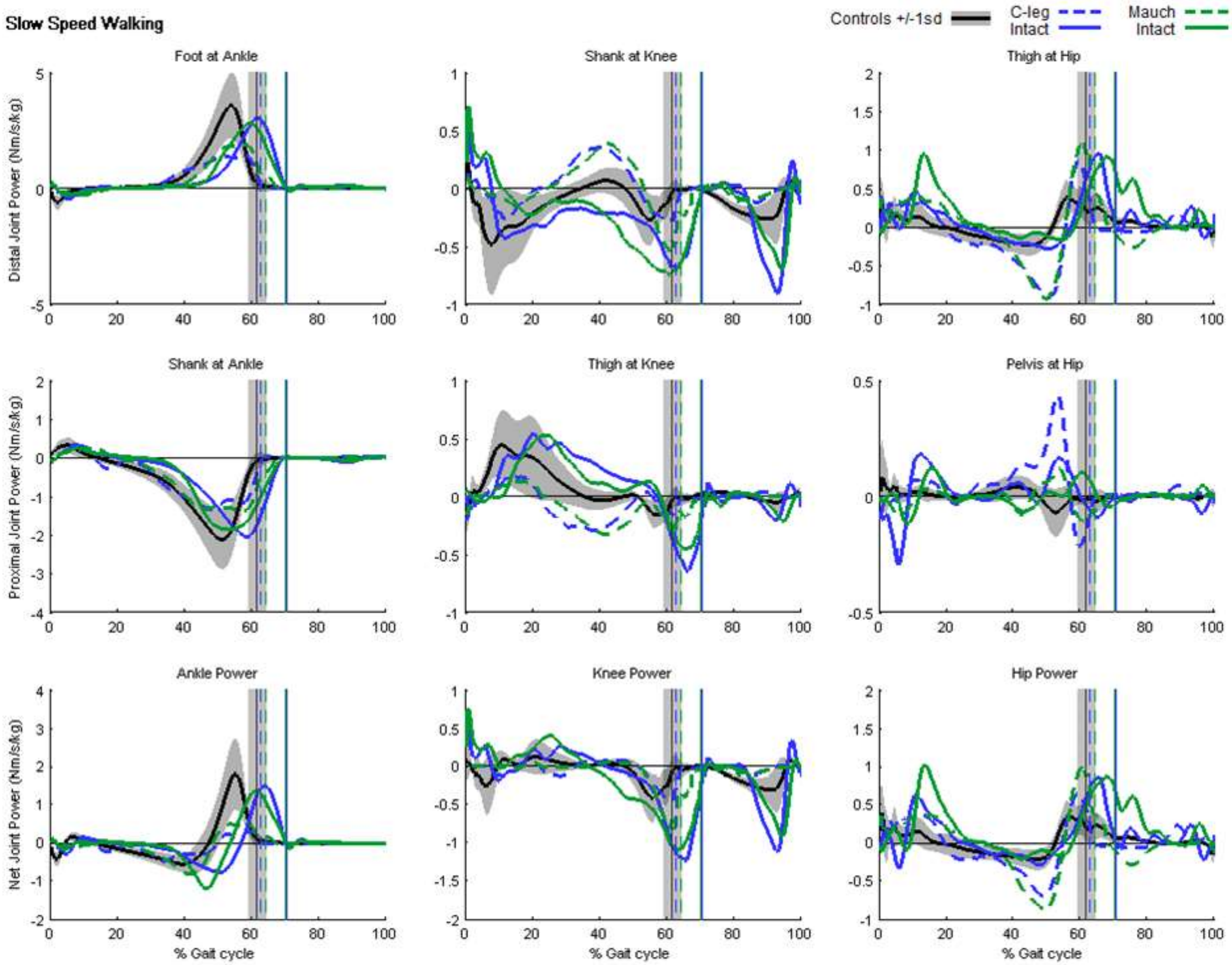
Slow Speed Walking
Controls +/-1sd
C-leg
Intact
Mauch
Intact
Foot at Ankle
Shank at Knee
Thigh at Hip
Distal Joint Power (Nm/s/kg)
Shank at Ankle
Thigh at Knee
Pelvis at Hip
Proximal Joint Power (Nm/s/kg)
Ankle Power
Knee Power
Hip Power
Net Joint Power (Nm/s/kg)
% Gait cycle
% Gait cycle
% Gait cycle

Figure S2.2(B)

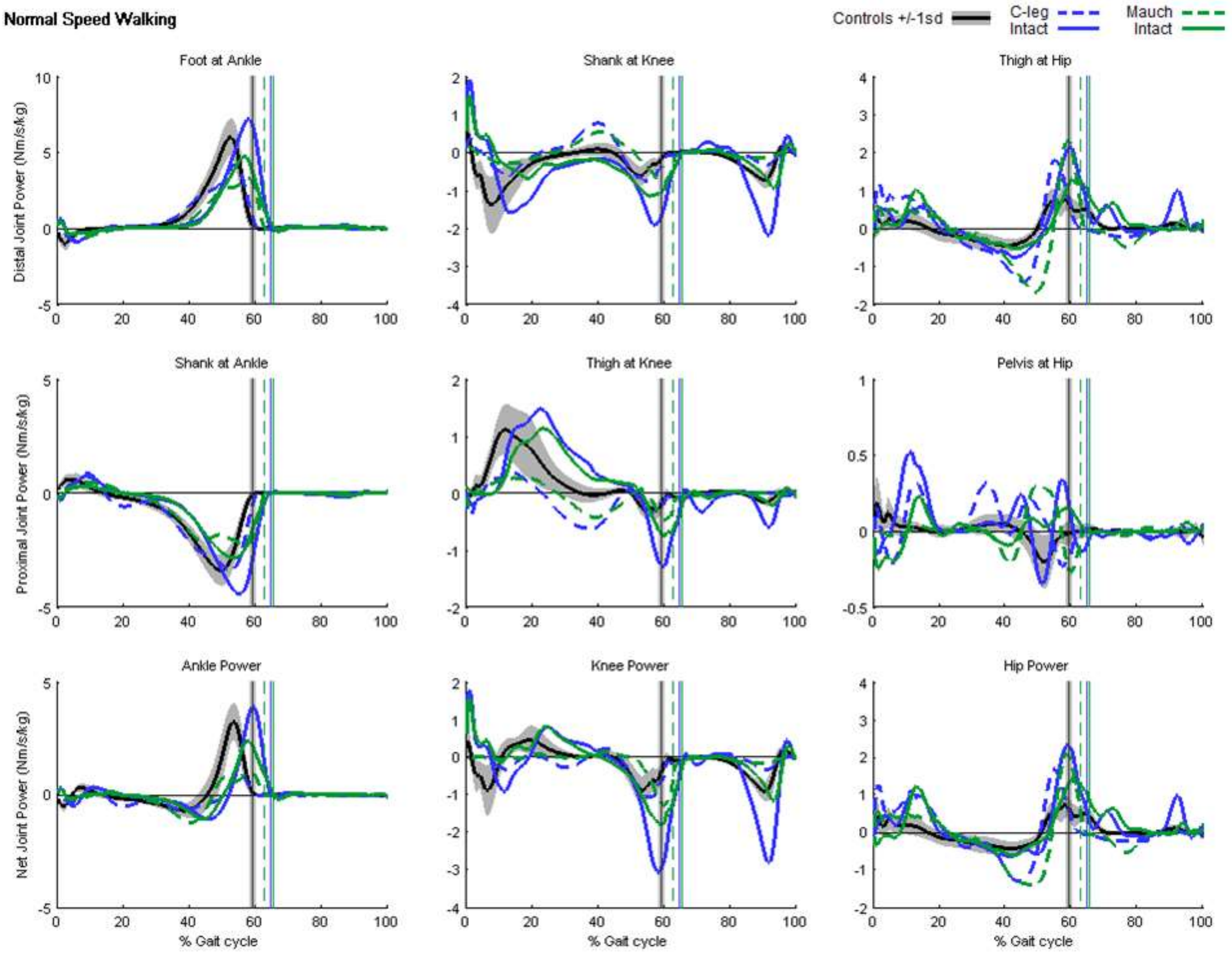

Normal Speed Walking
Controls +/-1sd
C-leg
Intact
Mauch
Intact
Foot at Ankle
Shank at Knee
Thigh at Hip
Distal Joint Power (Nm/s/kg)
Shank at Ankle
Thigh at Knee
Pelvis at Hip
Proximal Joint Power (Nm/s/kg)
Ankle Power
Knee Power
Hip Power
Net Joint Power (Nm/s/kg)
% Gait cycle
% Gait cycle
% Gait cycle

Figure S2.2(C)

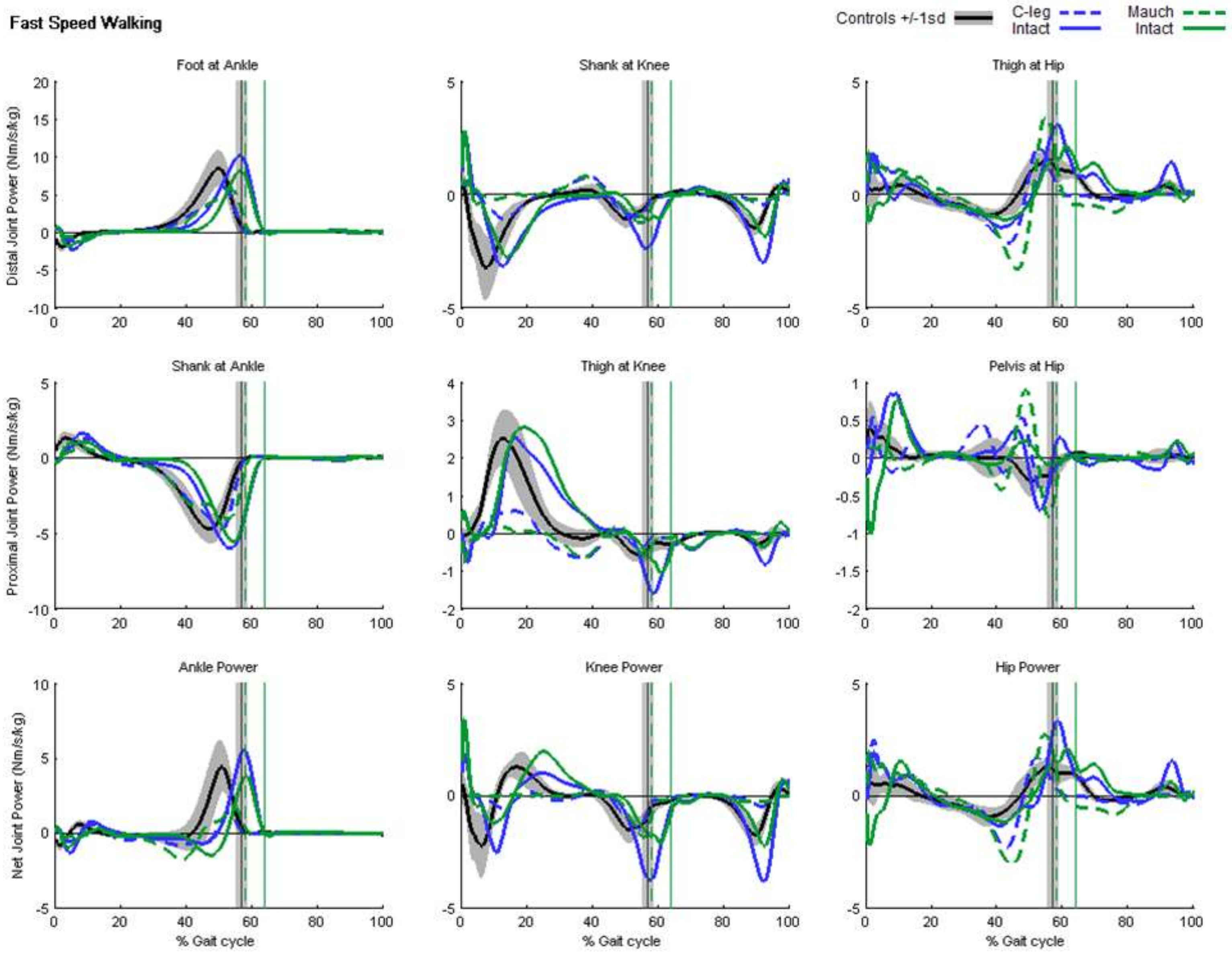

Fast Speed Walking
Controls +/-1sd
C-leg
Intact
Mauch
Intact
Foot at Ankle
Shank at Knee
Thigh at Hip
Distal Joint Power (Nm/s/kg)
Shank at Ankle
Thigh at Knee
Pelvis at Hip
Proximal Joint Power (Nm/s/kg)
Ankle Power
Knee Power
Hip Power
Net Joint Power (Nm/s/kg)
% Gait cycle
% Gait cycle
% Gait cycle
