# Supplementary material for: Development of a Mechanistic Hypothesis Linking Compensatory Biomechanics and Stepping Asymmetry during Gait of Transfemoral Amputees
Source: Appl Bionics Biomech. 2019 Feb 3;2019:4769242. doi: 10.1155/2019/4769242 (PMC6378070; doi:10.1155/2019/4769242)

S3 – External work rate on CoM and Internal work rate of joints

S3 Fig 1. External work on the body CoM during the gait cycle. Data are shown for amputee with C-Leg prosthesis (A=slow, B=normal, C=fast) and amputee with Mauch prosthesis (D=slow, E=normal, F=fast). In each panel the first column of plots shows gravity work rate (power) on center of mass (CoM), and 2nd column shows impulse work rate (power) on CoM. The first row shows Case 1 where intact limb is push-off limb (blue) and Case 2 where push-off limb is prosthesis (red line). Means for control subjects (N=14) is shown by dark solid lines with shaded boundaries that represent +/- 1 standard deviation from the mean at each % cycle. Vertical solid lines represent toe off time of the ipsilateral limb, and the dashed vertical lines represent heel strike of the contralateral limb, and the shaded boundaries represent +/- 1 standard deviation in event time. The horizontal axis shows 0-100% gait cycle.

S3 Fig 2. Internal work of joints during the gait cycle. Data are shown for amputee with C-Leg prosthesis (A=slow, B=normal, C=fast) and amputee with Mauch prosthesis (D=slow, E=normal, F=fast). In each panel the first column of plots shows ankle power, and 2nd column shows knee power, and the 3rd column shows hip power. The first row shows Case 1 where intact limb is push-off limb (blue) and Case 2 where push-off limb is prosthesis (red line). Means for control subjects (N=14) is shown by dark solid lines with shaded boundaries that represent +/- 1 standard deviation from the mean at each % cycle. Vertical solid lines represent toe off time of the ipsilateral limb, and the dashed vertical lines represent heel strike of the contralateral limb, and the shaded boundaries represent +/- 1 standard deviation in event time. The horizontal axis shows 0-100% gait cycle.

Figure S3.1(A)

External work on CoM for C-Leg at Slow speed

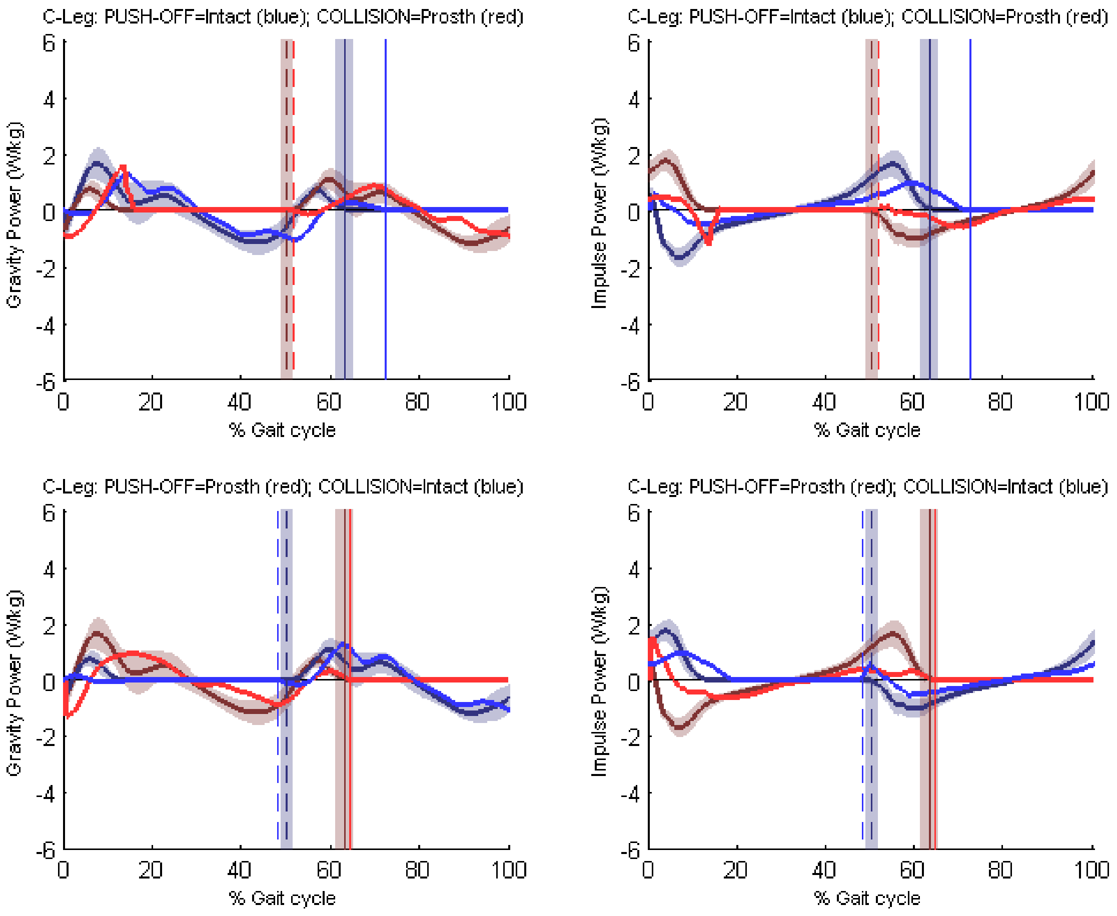

Figure S3.1(B)

External work on CoM for C-Leg at Normal speed

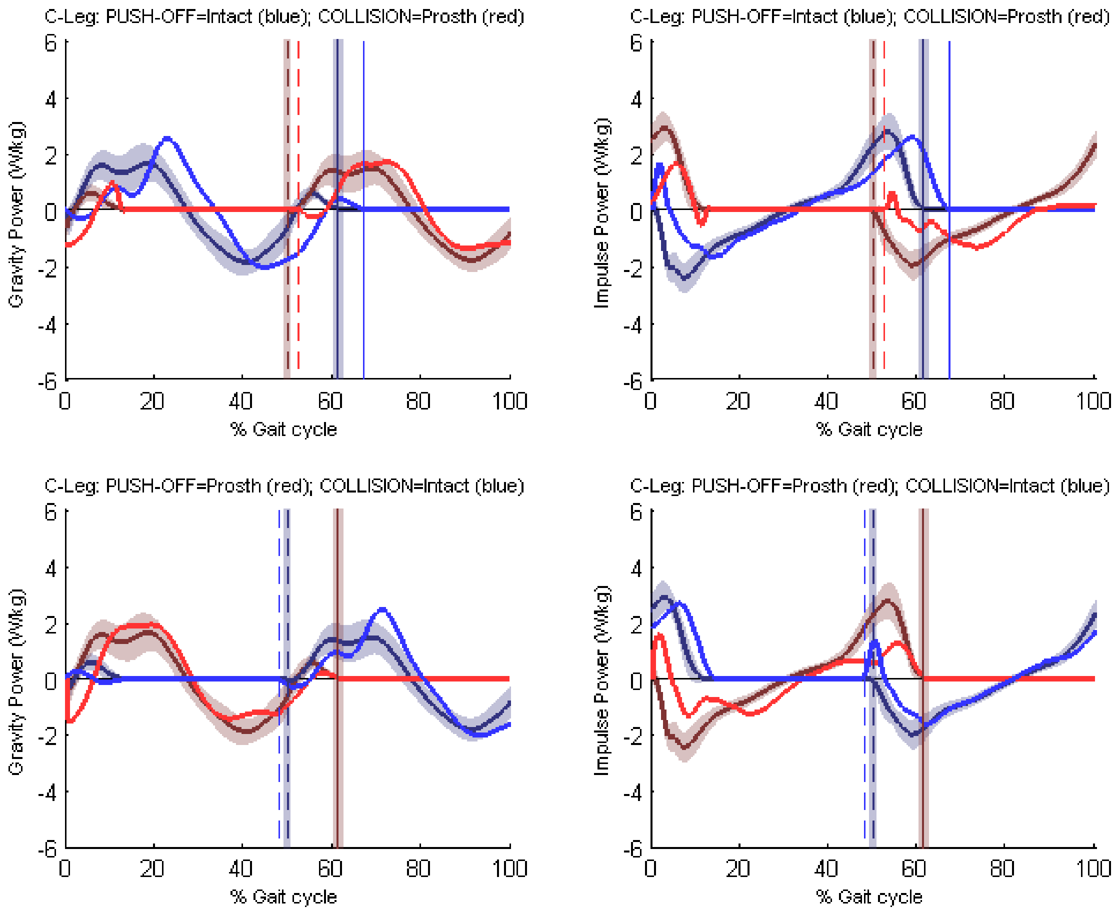

Figure S3.1(C)

External work on CoM for C-Leg at Fast speed

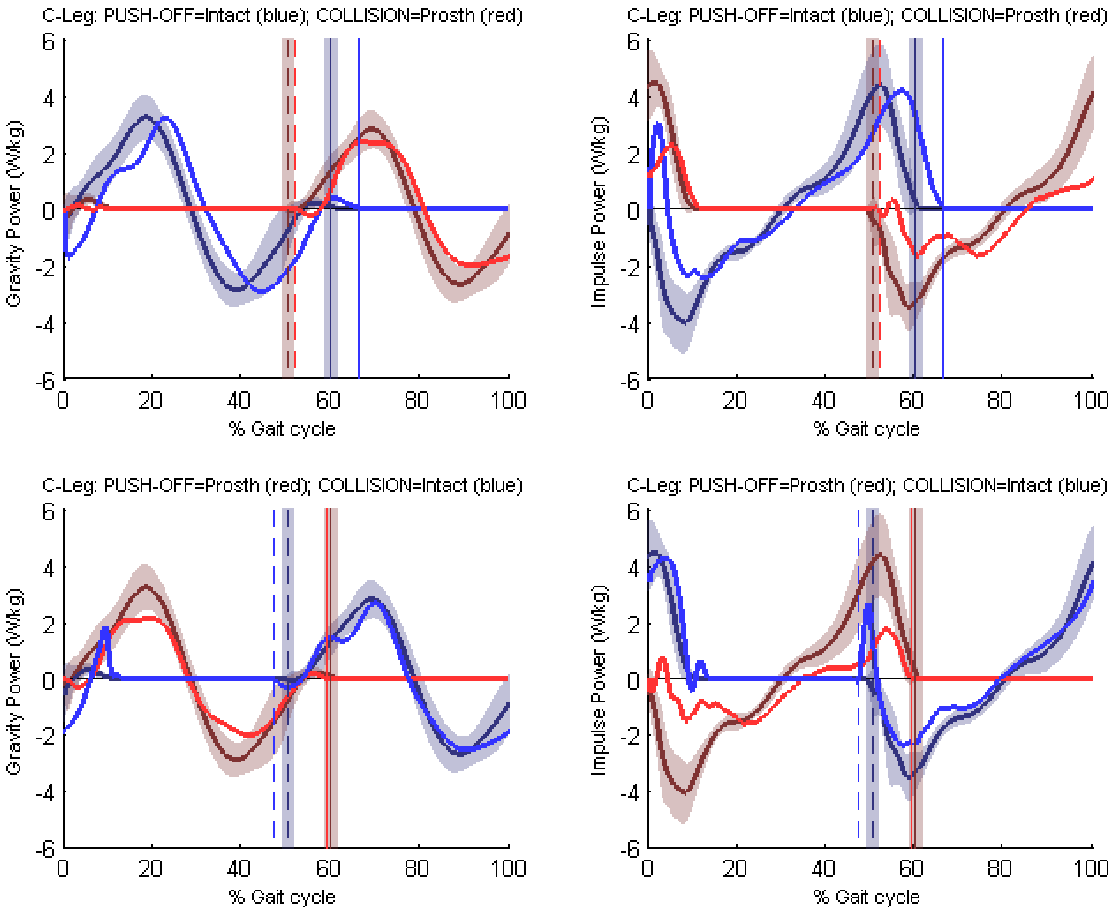

Figure S3.1(D)

External work on CoM for Mauch knee at Slow speed

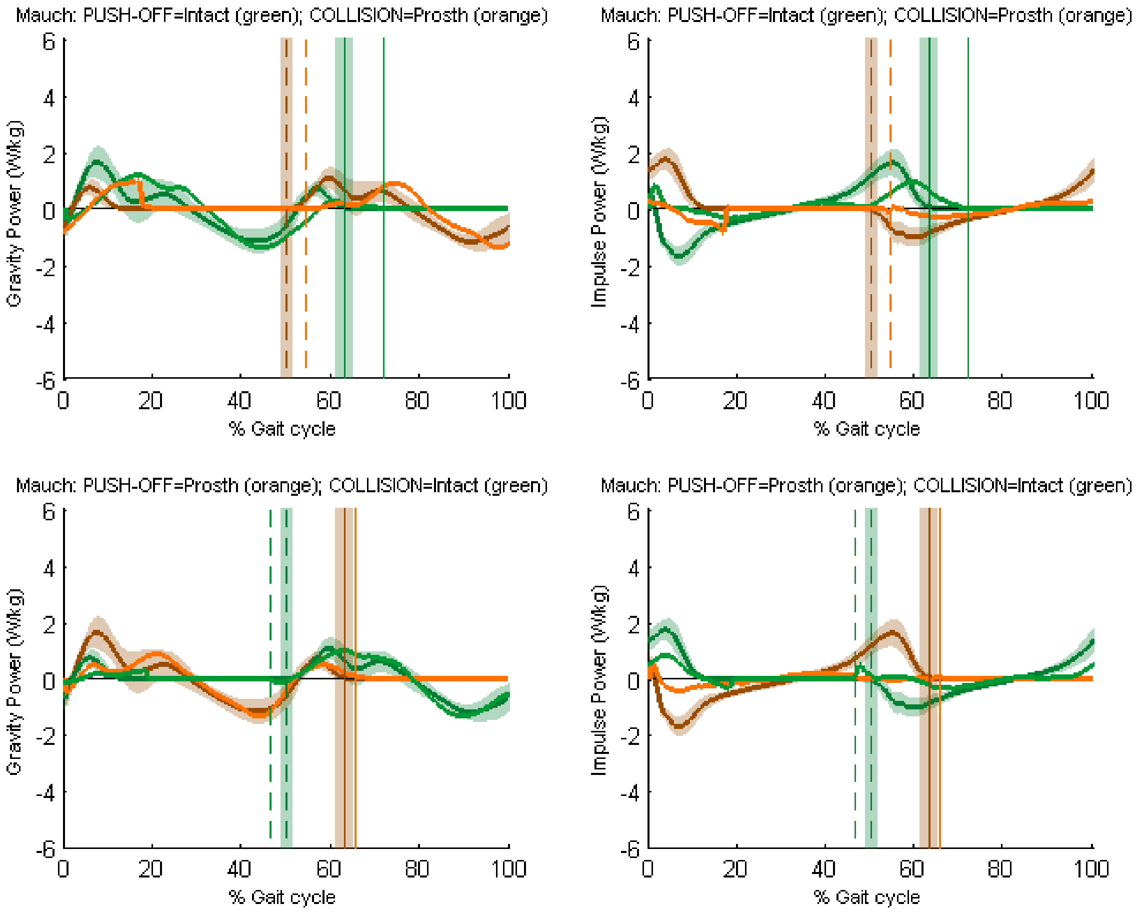

Figure S3.1(E)

External work on CoM for Mauch knee at Normal speed

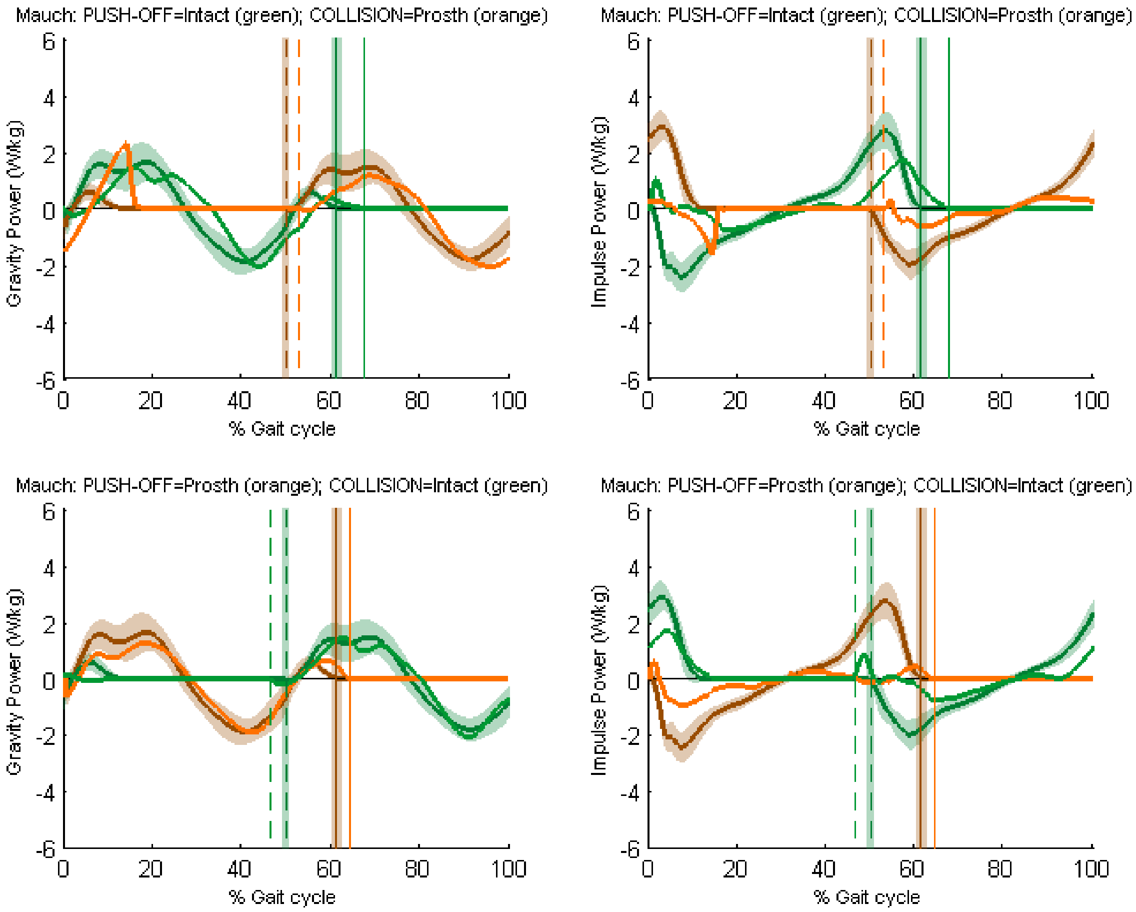

Figure S3.1(F)

External work on CoM for Mauch knee at Fast speed

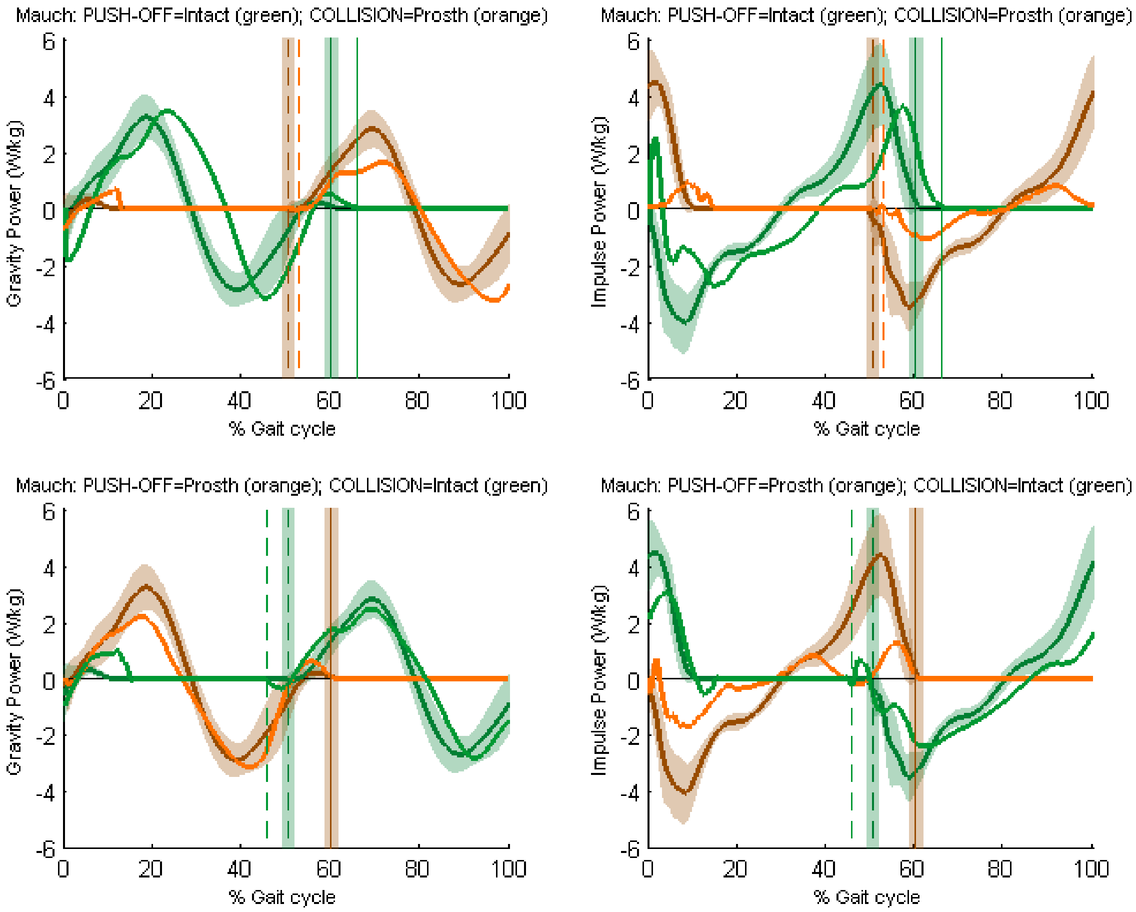

Figure S3.2(A)

Internal joint work for C-Leg at Slow speed

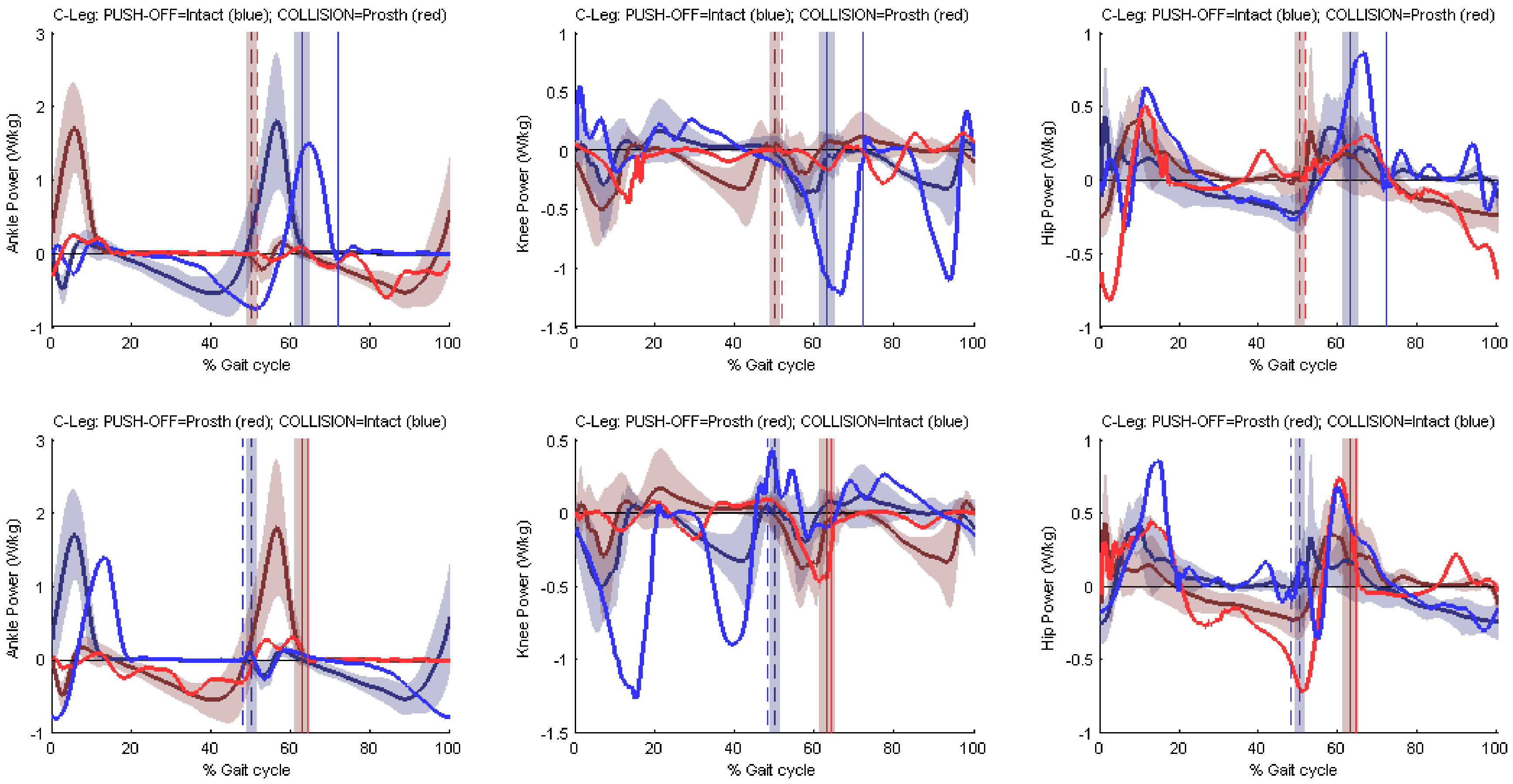

Figure S3.2(B)

Internal joint work for C-Leg at Normal speed

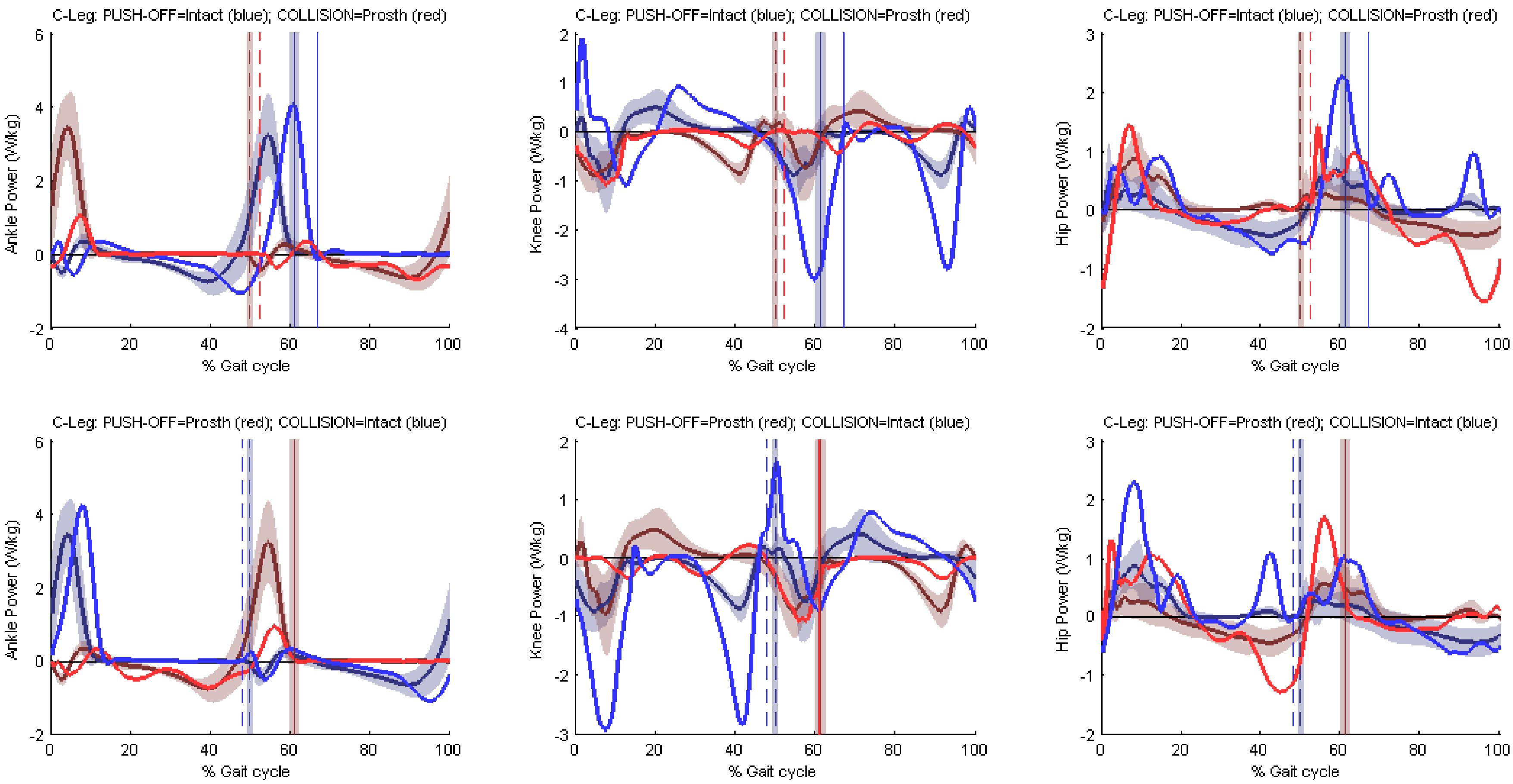

Figure S3.2(C)

Internal joint work for C-Leg at Fast speed

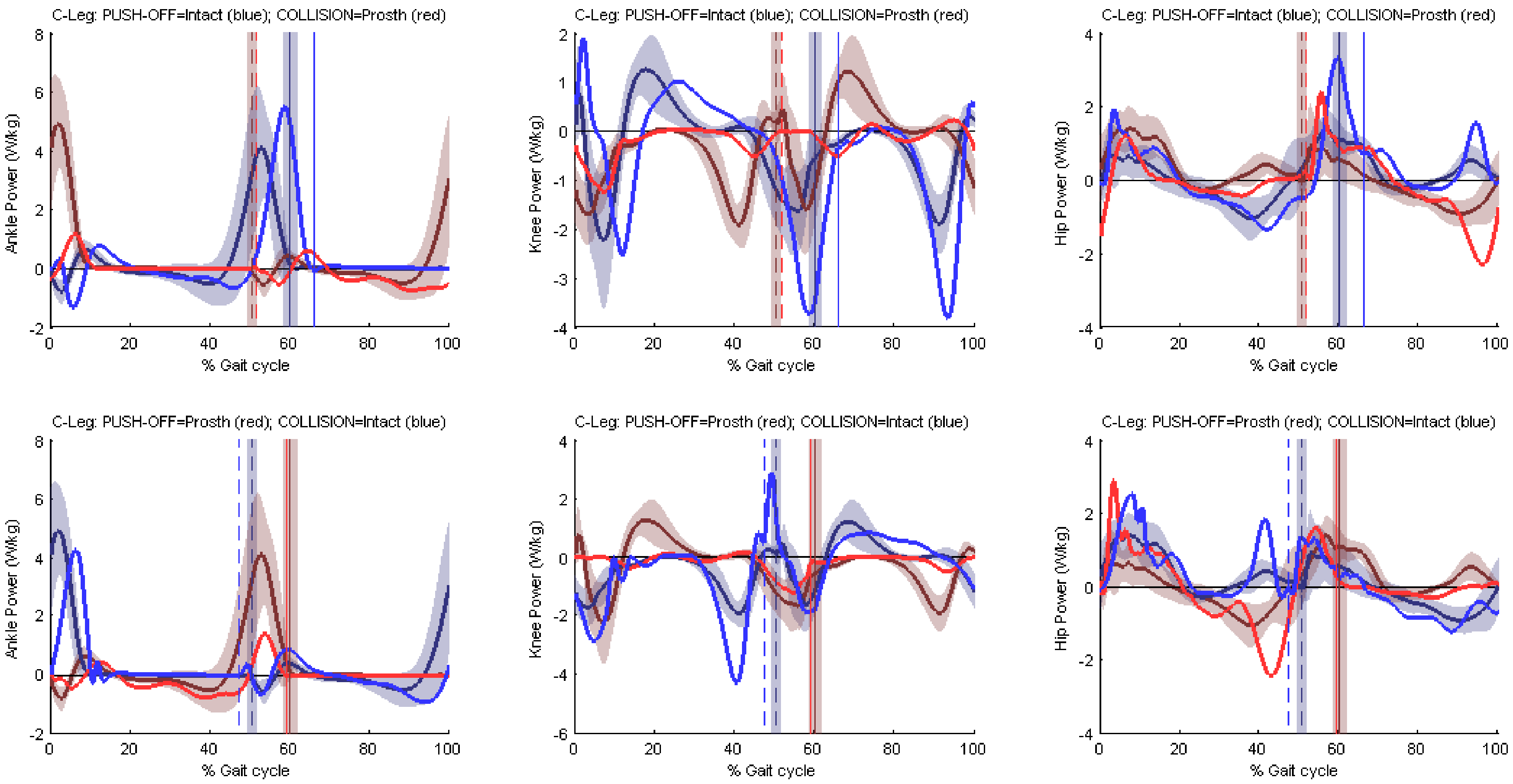

Figure S3.2(D)

Internal joint work for Mauch knee at Slow speed

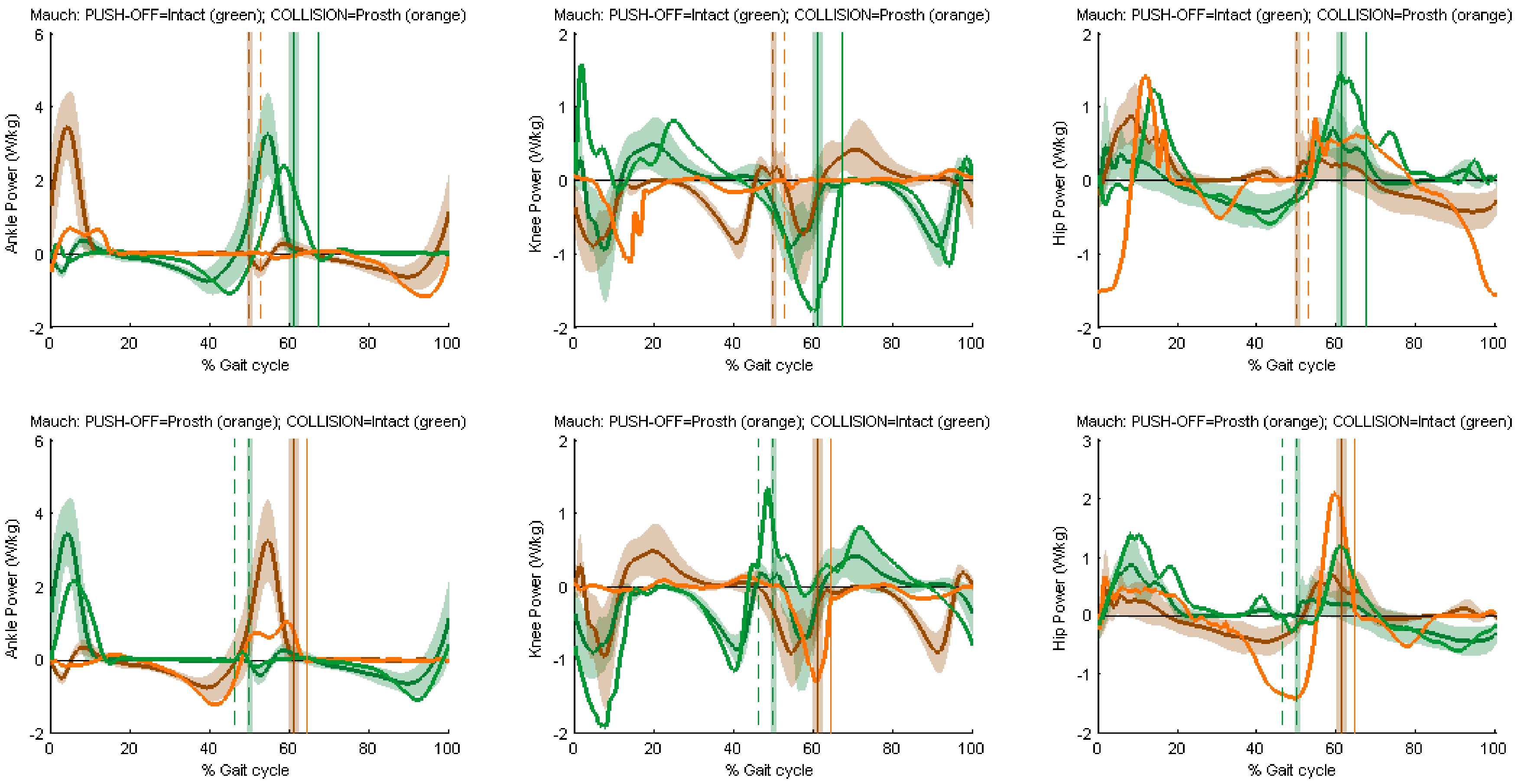

Figure S3.2(E)

Internal joint work for Mauch knee at Normal speed

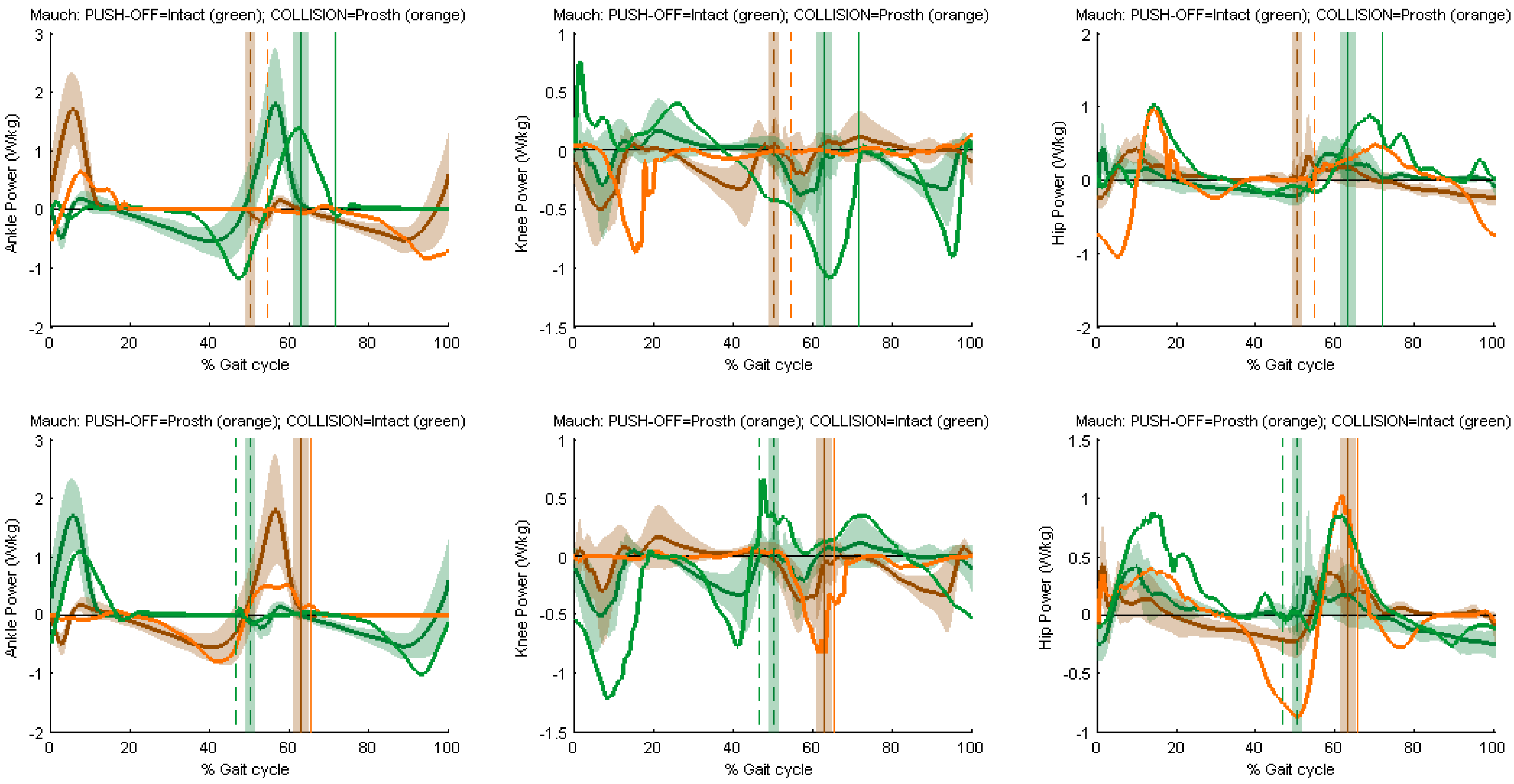

Figure S3.2(F)

Internal joint work for Mauch knee at Fast speed

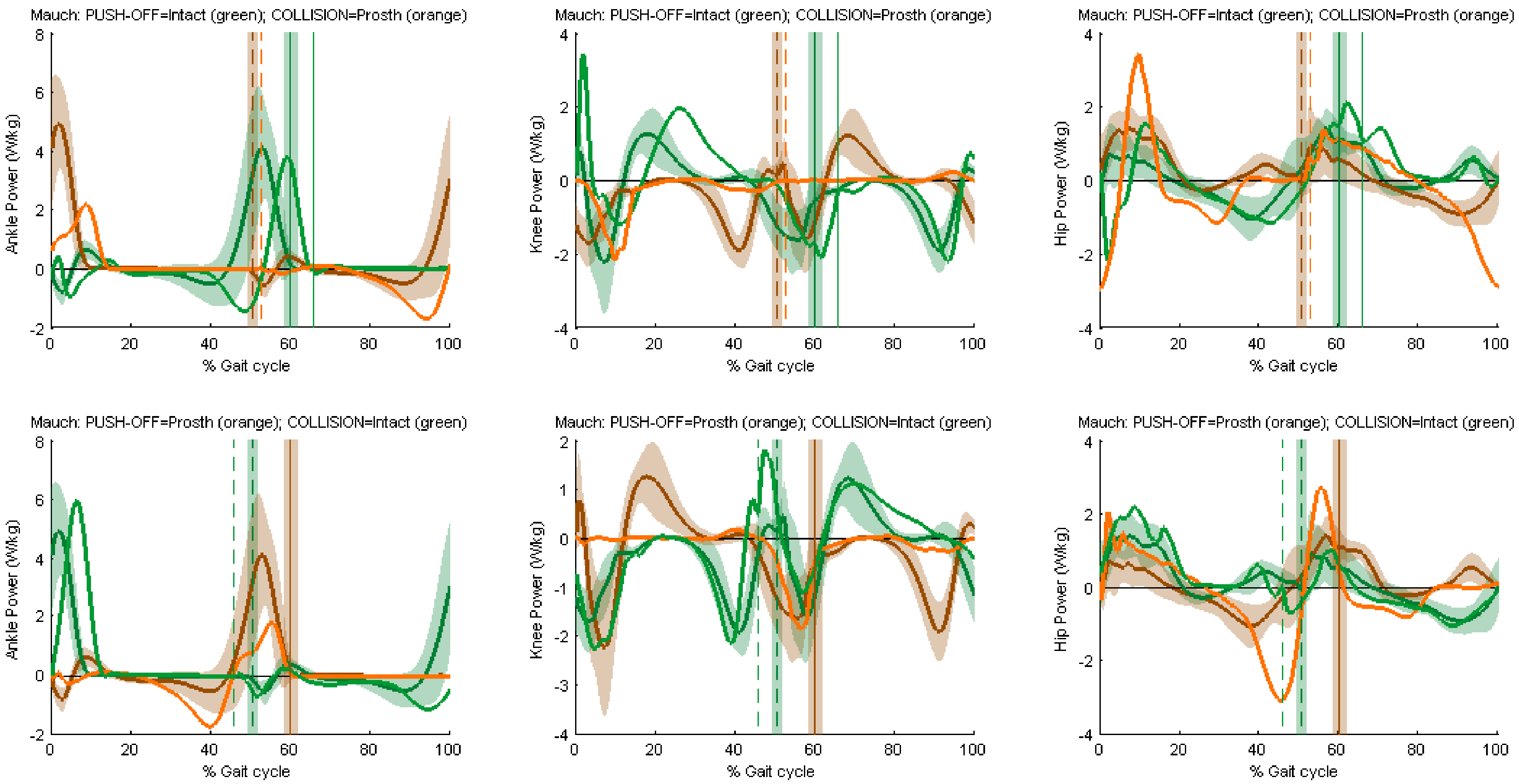

Supplement: Supplementary 3 — S3: external and internal work rate. These graphs show results for the 0-100% gait cycle for the intact limb and for the prosthetic limb, at each of the three speeds, for each of the two amputees. Figure S3.1 contains external work rate (gravity and impulse) for healthy controls with data for two amputees (C-Leg user and Mauch user) superimposed. Figure S3.2 contains internal work rate (ankle, knee, and hip) for healthy controls with data for two amputees (C-Leg user and Mauch user) superimposed. [file 4769242.f3.pdf]
